# The labor market costs of work-related stress: A longitudinal study of 52 763 Danish employees using multi-state modeling

**DOI:** 10.5271/sjweh.4131

**Published:** 2024-03-01

**Authors:** Jacob Pedersen, Brian Krogh Graversen, Kristian Schultz Hansen, Ida Elisabeth Huitfeldt Madsen

**Affiliations:** 1 National Research Centre for the Working Environment, Copenhagen, Denmark.; 2 National Institute of Public Health, Copenhagen, Denmark.

**Keywords:** ELMA, occupational health, sickness absence, unemployment, work

## Abstract

**Objective:**

Work-related stress is an important public health concern in all industrialized countries and is linked to reduced labor market affiliation and an increased disease burden. We aimed to quantify the labor market costs of work-related stress for a large sample of Danish employees.

**Methods:**

We linked four consecutive survey waves on occupational health and five national longitudinal registers with date-based information on wage and social benefits payments. From 2012 to 2020, we followed survey participants for two year-periods, yielding 110 559 person-years. We identified work stress by combining three dichotomous stress indicators: (i) self-perceived work stress, (ii) Cohen 4-level perceived stress scale, and (iii) job strain. Using the multi-state expected labor market affiliation (ELMA) method, we estimated the labor market expenses associated with work-related stress.

**Results:**

Of the employees, 26–37% had at least one work-stress indicator. Men aged 35–64 years and women aged 18–64 years with work-related stress had up to 81.6 fewer workdays and up to 50.7 more days of sickness absence during follow-up than similarly aged men without work stress. The average annual work absenteeism loss per employee linked to work-related stress was €1903 for men and €3909 for women, corresponding to 3.3% of men’s average annual wages and 9.0% of women’s average annual wages, respectively. The total annual expenses were €305.2 million for men and €868.5 million for women.

**Conclusion:**

Work-related stress was associated with significant labor market costs due to increased sickness absence and unemployment. The prevention of work-related stress is an important occupational health concern, and the development of effective interventions should be given high priority.

Work-related stress profoundly affects labor market affiliation in terms of increased risk of employees experiencing sickness absence (1–7), lowered probability of returning to work (8), and increased risk of an early exit from the labor market (9–11). Nevertheless, almost all economic and epidemiologic studies on work-related stress include only a single labor market outcome (12), such as the risk of sickness absence. Studies investigating the impact of multiple labor outcomes and their interconnectivity are rare (8), thereby omitting essential knowledge concerning recurrent sickness absence leading to decreased work participation, unemployment, and early retirement (8, 13). Moreover, translating the findings into real-world contexts such as costs can be challenging for companies and employers facing the complex behavior of sick listings among employees.

Economic studies that deal with work-related stress and its associated labor market consequences often use aggregated portions or results from the research literature to make assumptions on costs, eg, 11 of 15 studies in the review by Hassard et al (14). Such studies may have high macroeconomic relevance, but again, not necessarily precisely the type of specific information applicable to the individual employer or employee (14). In contrast, only a few studies use information from individual wage payments when estimating costs per work-related stress case. One Australian incidence-based study, one Swiss prevalence-based study, and one incidence-based study from the United Kingdom estimated that work-related stress costs society €124–529 per afflicted employee (14), with an average 2014 exchange rate of US$1 = €0.7541 (15). The respective annual costs accumulated to €3.0 and €4.1 billion per year (16–18). Additionally, the Swiss study reported that the highest cost of work-related stress concerns sickness absence wages (59.9%), followed by medical service use (31.5%) and self-medication (8.6%) (17). However, these three studies are not directly comparable, as they include different definitions of work-related stress given by mental stress, anxiety, and depression. Moreover, the range of included healthcare and non-healthcare expenses differ and encompasses, eg, doctor visits, rehabilitation, tax loss, and insurance costs.

Principally, the work-related stress costs per employee may differ for many reasons, including country differences in the labor market system, the healthcare system, healthcare expenses, and non-healthcare expenses. Additionally, the methodological approach may influence the results. The top-down approach aggregates the national burden portion of a specific health problem concerning medical, sick leave, and value of life costs. In contrast, the bottom-up approach takes the estimated cost per case and extrapolates it to a national level. The bottom-up approach typically contains a higher variety of cost components per case or person than the top-down approach. However, the bottom-up approach relies on more detailed data sources, and the analysis may therefore be more time-consuming (14). The human capital approach assumes that reductions in employment of an employee reduce society’s production value by the reduction in working hours of the employee times the employee’s productivity per hour of work measured by the hourly wage rate (19).

This study aimed to quantify the labor market costs associated with work-related stress for a large sample of Danish employees. Utilizing the expected labor market affiliation (ELMA) method in a prospective study, we take a human capital and bottom-up approach to the societal cost of work-related stress concerning reduced work production value in terms of increased sickness absence and unemployment. However, since we can only estimate the actual production value lost while employees were absent from work, we use the term ‘costs of work absenteeism’ to describe the costs of any negative difference between the number of working days deduced from the analysis and the expected number of working days. The ELMA method has shown to be a well-founded analytical tool for analyzing multiple labor market outcomes while including the interconnectivity between multiple outcomes (8, 13, 20).

This study included three indicators of work-related stress: (i) self-perceived work-related stress, defined as the degree to which situations in one’s working life are appraised as stressful (21); (ii) Cohen’s four-level perceived stress scale (21); and (iii) job strain, defined as a combination of high quantitative demands and low influence (22). While the first two indicators concern work-related stress as reported by the employee, the third indicator, job strain, is a widely applied operationalization of psychosocial stressors, ie, potentially stressful situations at work (23). Job strain is likely to identify individuals who have not yet developed symptoms of stress or are unaware of their stress reactions.

## Methods

### Study design and source population

This longitudinal study analyses survey data on work-related stress from four successive waves of the Work Environment and Health in Denmark (WEHD) study conducted in 2012, 2014, 2016, and 2018 (3, 24). The WEHD surveys each contain a sample of 18–64 aged Danish employees. Details on the WEHD surveys are presented in the supplementary material, URL, part A. The WEHD data were linked to national registers (25), and WEHD responders were followed in registers for two years from the date of survey response. Individuals who responded to multiple waves were included for multiple follow-up periods.

The WEHD data were linked with five registers through Statistics Denmark: (i) the Danish labor market accountant (LMA), (ii) work absences (RoWA), (iii) education, (iv) emigration and immigration, and (v) the death register. We included data from 2010 until the end of 2020. LMA contains information on all major social benefits payments, including unemployment, sickness absence, disability pension, pension, and all wage payments reported to the tax authorities.

RoWA links the absence and employment register (FRAN) and the periods of absence register (FRPE), containing information about sickness absence spells from the first day of absence and employment information (3). RoWA contains records for all public and a large yearly sample of private employees, summing to about 37% coverage, including approximately 2600 private companies with ≥9 employees (26). The education register contains records of the highest education level completion for all Danes. The emigration and immigration register contains dates on all immigration and emigrations in Denmark. The death register includes death dates on all deceased Danes.

### Study sample and data preparation

We included all respondents from the four WEHD waves (N=85 271), totaling 124 859 follow-up periods. The study sample (N=52 763, and 75 537 follow-up periods) consisted of active employees not receiving a disability pension and with a follow-up linked only to the employer registered at the survey. The study sample was divided into six subsamples by sex and age: 18–34, 35–49, and 50–64 years. Since we included multiple survey waves, each employee may have had up to four follow-up periods. A detailed description of the sample selection process, including a flow chart, is presented in supplementary material B.

### Work-related stress

The study used one work-stress variable defined by combining three dichotomous (1=yes or 0=no) work-stress indicators: (i) self-perceived work stress, (ii) the Cohen four-item perceived stress scale modified to work stress, and (iii) job strain, high quantitative demands and low influence/job control at work. Each individual was classified as having either zero, one, any combination of two, or all three work-stress indicators during a follow-up period. For additional details on the three work-stress indicators, see supplementary material C.

### Covariates and weights

The analysis included nine covariates previously used in studies about work-related stress in relation to long-term sickness absence and work disability (3, 13). The covariates were associated with adverse health outcomes, possibly through selection, eg, selection into part-time work, or through causation, eg, smoking and sickness absence.

Five variables were included from WEHD: (i) body mass index (BMI, kg/m^2^) (underweight: BMI<18.0; normal weight: 18.5≤BMI< 25.0; overweight: 25.0≤BMI<29.9; and obese: BMI≥29.9). (ii) Smoking (yes: “daily” and “sometimes”; no: “prior smokers” and “never”). (iii) Alcohol consumption, defined as the number of items (15 ml of pure alcohol) per week (none; moderate: 1–9; high: ≥10). (iv) Physical activity “How much time on average do you use on each of the following physical activities in the last year?” as “exercise, heavy gardening or fast walking/cycling where you sweat and getting short of breath?” with the dichotomizing of the answering range (yes: >4, 2–4, and <2 hours/week; no: “Does not practice this activity” and missing). (v) Disease treatment – dichotomously defined as whether the individual has had treatment for one of the following diseases (no/yes): depression, asthma, diabetes, atherosclerosis or blood clot in the heart, blood clot in the brain (cerebral hemorrhage), cancer, back disease, migraine, or other long-term disease. (vi) Working time arrangement, ranging by the number of hours recorded at the follow-up starting state (low: 0–64%; medium: 65–94%; full-time: ≥95%) standardized and compared to a norm working day of 7.4 hours included from the LMA register. (vii) Employment sector (private/public) from the FRAN register. (viii) Highest accomplished education (low/middle/high) from the education registers. The variable (ix) “number of survey waves” was constructed to account for the number of WEHD survey waves the individual had attended –“1 of 4”, “2 of 4”, “3 of 4”, and “4 of 4”. Only variable (viii) was allowed to change during the follow-up period, while the remaining variables were updated only at the start of each individual follow-up period.

### Labor market affiliation

**Figure 1:**
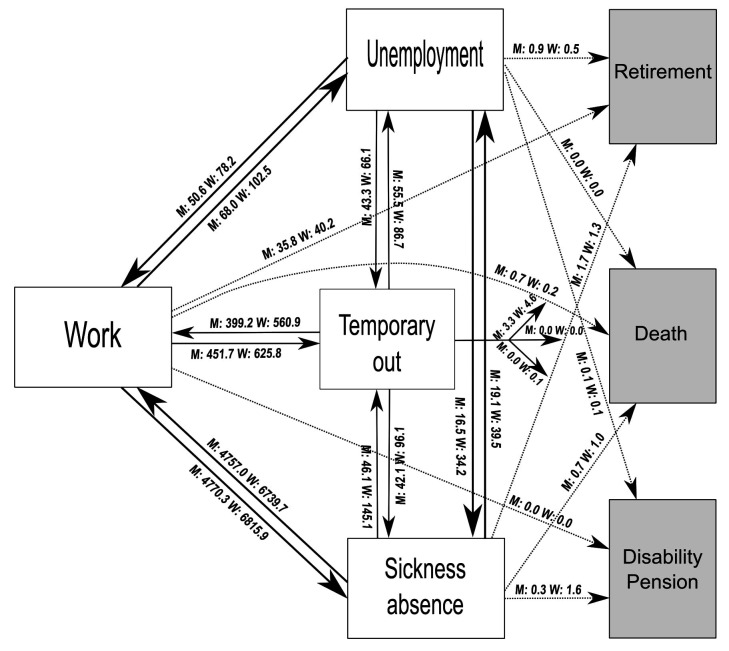
The multi-state labor market affiliation model with boxes representing labor states and arrows representing transitions. The lines represent transitions between the recurrent and absorbing states, and the numbers show the general flow in events per 1000 person-years.

The labor market affiliation was modeled by seven mutually exclusive labor market states – four recurrent states (work, sickness absence, unemployment, temporary out) and three absorbing states (retirement, disability pension, death) as illustrated in figure 1. The modeling was based on a “long format” arrangement (27) of the longitudinal linkage of the LMA and RoWA. Absorbing states were prioritized over recurrent states, and prior states overwrite subsequent vacation time; moreover, neither the LMA nor RoWA contains any registration of leisure time. If a record contains multiple payments such as wage and sickness absence benefits, we prioritized the payment with the most recorded hours as the labor market state. The follow-up started in any of the four recurrent states.

The follow-up period was censored at the first occurrence of either the end of the two-year follow-up, if reaching the age of 65, when a new follow-up period started for the same individual (because the individual had been interviewed again in a subsequent survey round), or if a new employer-id was registered, whichever came first.

Supplementary material D contains a detailed description of the states of the model, including a short introduction to the Danish labor market and social system.

### Statistical analysis

The study used the ELMA method developed by Pedersen et al (8), which relies on estimated transition probabilities between the possible states of the multi-state model. The ELMA incorporates time-invariant variables, time-varying variables, and weights. The ELMA uses Cox proportional hazard regression for establishing time-dependent transition probabilities for each covariate while incorporating modern survival terms such as left and right censoring, time truncation, recurrent events, and competing events management while fulfilling a Markov assumption (28). Using numerical integration, ELMA converts complex patterns of state-conditioned transition probabilities into overall state duration estimates (29, 30) before conducting variance analysis on the duration estimates to find the variable-specific contributions (8, 13, 31).

For each subsample of sex and age groups, we estimated the time-dependent baseline transition probability for each of the 24 arrows in the multi-state model (see figure 1), using employees with no work-stress indicators as the reference group. Then, we estimated the transition probabilities for the non-reference values by adjusting the baseline transition probabilities with estimates derived from corresponding transition-specific Cox proportional hazard regressions. Based on the Chapman–Kolmogorov equation, we calculated the state probabilities and estimated the area under the transition and state probabilities. Then, we combined the area estimates to express the expected time spent in each of the seven states during the 730 days of follow-up.

We used 1000 normally distributed random resamples of the area estimates to produce the state duration 95% confidence intervals (CI). All variables, except the work-stress indicator variable, were incorporated into the model as inverse probability weights, which we multiplied with the weights from RoWA.

For sensitivity analysis purposes, we compared the ELMA results with crude estimates on the state durations. The crude estimates were calculated by the sum of days for all employees within the state during follow-up, divided by the total number of employees – grouped by sex and age.

### Cost estimation

We estimated the work-stress-related costs regarding work absenteeism, sickness absence, unemployment, and temporary out. This was done using date-based information on individual gross wage payments and working hours from the LMA register. The individual wage payments were standardized to hourly payments, using the Danish norm of 7.4 working hours per day (37-hour working week) – truncating extreme payments to a minimum limit of €6.72 per hour (DKK50) and a maximum limit of €268.63 per hour (DKK2000). Any missing information on hourly wages was imputed by regression using baseline information on sex, age group, education level, sector, and industry group, and then all wages were transformed into a 2022 price level. We then estimated the state-specific annual cost per employee by multiplying the individual standardized hourly wage payments with 7.4 hours per day and additionally with the state-specific durations per year deduced from the ELMA analysis – estimated as reduced or increased number of days per year. We made the cost estimates representative of Danish employees by multiplying them with weights retained from the WEHD data. Then, we estimated the state-specific annual average cost per employee and yearly total costs by sex and age groups with corresponding 95% CI.

All results on costs are presented at the 2022 price level, as we adjusted all wages using the sex and age-specific consumer price index from Statistics Denmark (32).

For sensitivity analysis purposes, we compared the ELMA cost results with (i) the crude estimates, (ii) the cost with the inclusion of part-time wages, and (iii) the cost using each of the three stress indicators separately (supplementary material E). Supplementary material F contains analyses on the hypothetical reduction potential regarding the total annual value of work absenteeism and sickness absence and a top-down estimation of the society costs. The study was inspired by the Consensus Health Economic Criteria (CHEC) list for securing the methodological quality of the study (33).

## Results

Table 1 shows that the study sample includes 52% women (N=23 616) and 48% men (N=22 120). Moreover, more women than men experienced work-related stress concerning the number of work-stress indicators.

**Table 1:** Descriptive characteristics of the study population at baseline of the first follow-up period of the individual employee.

		Men (age in years)						Women (age in years)				
		18–34		35–49		50–64		18–34		35–49		50–64
		N (%)		N (%)		N (%)		N (%)		N (%)		N (%)
TOTAL		3730 (17)		8593 (39)		9797 (44)		5226 (17)		12437 (41)		12980 (42)
Self-perceived stress	No	3331 (89)		7590 (88)		8798 (90)		4344 (83)		10421 (84)		11051 (85)
	Yes	399 (11)		1003 (12)		999 (10)		882 (17)		2016 (16)		1929 (15)
Cohen four-item stress	No	3073 (82)		7096 (83)		8335 (85)		4024 (77)		9916 (80)		10601 (82)
	Yes	657 (18)		1497 (17)		1462 (15)		1202 (23)		2521 (20)		2379 (18)
Job strain	No	3244 (87)		7308 (85)		8561 (87)		4320 (83)		10544 (85)		11080 (85)
	Yes	486 (13)		1285 (15)		1236 (13)		906 (17)		1893 (15)		1900 (15)
Number of work-stress indicators	0 of 3	2633 (71)		6001 (70)		7270 (74)		3292 (63)		8233 (66)		8933 (69)
	1 of 3	743 (20)		1675 (19)		1624 (17)		1120 (21)		2480 (20)		2384 (18)
	2 of 3	263 (7)		641 (7)		636 (6)		572 (11)		1222 (10)		1165 (9)
	3 of 3	91 (2)		276 (3)		267 (3)		242 (5)		502 (4)		498 (4)
Body mass index	Underweight	20 (1)		16 (0)		20 (0)		162 (3)		176 (1)		190 (1)
	Normal weight	1900 (51)		3265 (38)		3337 (34)		3055 (58)		6698 (54)		6732 (52)
	Overweight	1132 (30)		3598 (42)		4529 (46)		1019 (19)		3235 (26)		3714 (29)
	Obese	346 (9)		1315 (15)		1596 (16)		550 (11)		1775 (14)		1828 (14)
	Not available	332 (9)		399 (5)		315 (3)		440 (8)		553 (4)		516 (4)
Smoking	Nonsmoker	2631 (71)		6622 (77)		7595 (78)		3923 (75)		9925 (80)		10156 (78)
	Smoker	787 (21)		1591 (19)		1931 (20)		942 (18)		2082 (17)		2448 (19)
	Not available	312 (8)		380 (4)		271 (3)		361 (7)		430 (3)		376 (3)
Weekly alcohol consumption	None	646 (17)		1316 (15)		995 (10)		1540 (29)		3158 (25)		2285 (18)
	Moderate	1333 (36)		3310 (39)		2985 (30)		2145 (41)		5848 (47)		5353 (41)
	High	1442 (39)		3584 (42)		5549 (57)		1182 (23)		2986 (24)		4963 (38)
	Not available	309 (8)		383 (4)		268 (3)		359 (7)		445 (4)		379 (3)
Physical activity	No	2052 (55)		4778 (56)		5639 (58)		3091 (59)		7493 (60)		7914 (61)
	Yes	1678 (45)		3815 (44)		4158 (42)		2135 (41)		4944 (40)		5066 (39)
Disease treatment	No	1345 (36)		3200 (37)		3197 (33)		1666 (32)		4410 (35)		4269 (33)
	Yes	292 (8)		1090 (13)		1637 (17)		667 (13)		2199 (18)		2358 (18)
	Not available	2093 (56)		4303 (50)		4963 (51)		2893 (55)		5828 (47)		6353 (49)
Status time arrangement	≥95% of 37 hours/week	3009 (81)		7483 (87)		8397 (86)		3606 (69)		8208 (66)		8370 (64)
	65–94% of 37 hours/week	559 (15)		1019 (12)		1274 (13)		1322 (25)		3991 (32)		4306 (33)
	0–64% of 37 hours/week	162 (4)		91 (1)		126 (1)		298 (6)		238 (2)		304 (2)
Employment sector	Private	2375 (64)		5791 (67)		6034 (62)		1752 (34)		3815 (31)		3342 (26)
	Public	1355 (36)		2802 (33)		3763 (38)		3474 (66)		8622 (69)		9638 (74)
Highest educational level	Low	395 (11)		882 (10)		1517 (15)		289 (6)		710 (6)		1714 (13)
	Middle	1552 (42)		3413 (40)		4459 (46)		1847 (35)		4418 (36)		5171 (40)
	High	1768 (47)		4245 (49)		3741 (38)		3054 (58)		7269 (58)		6054 (47)
	Not available	15 (0)		53 (1)		80 (1)		36 (1)		40 (0)		41 (0)
Number of survey waves	1 of 4	2912 (78)		6194 (72)		7307 (75)		3961 (76)		8605 (69)		9300 (72)
	2 of 4	559 (15)		1445 (17)		1622 (17)		866 (17)		2222 (18)		2355 (18)
	3 of 4	150 (4)		434 (5)		408 (4)		248 (5)		699 (6)		601 (5)
	4 of 4	109 (3)		520 (6)		460 (5)		151 (3)		911 (7)		724 (6)

Figure 1 illustrates the multi-state labor market model, with arrows representing the possible transitions. Transitions from work to sickness absence and back were most frequent, with over 6700 events per 1000 person-years for women and over 4700 events per 1000 person-years for men. The second most frequent transitions were between temporary out and work. Transitions to the absorbing states were infrequent, except for retirement from work. The model contains 110 559 person-years of follow-up.

Table 2 presents the ELMA results. To find the expected days for the individual or the combination of work stress indicators, you add (+) or subtract (-) the number of days presented for one, two, or three work stress indicators to the reference value. For men aged 35– 64 years and women aged 18–64 years, an overall pattern can be seen; for an increasing number of work stress indicators, the number of work days decreased, while the number of sickness absence days and unemployment days increased.

**Table 2:** Estimated labor market affiliation (ELMA) results given by the expected number of days during the two-year follow-up period spent in the four recurrent labor market states stratified by sex and age groups. Reference value showing the expected days and the additional or subtracted days (+/-) for employees with 1–3 indicators of work stress. [Ref= Reference value; CI=confidence interval].

Number of work-stress indicators			Work				Sickness absence				Unemployment				Temporary out		
			ELMA ^a^		Crude		ELMA ^a^		Crude		ELMA ^a^		Crude		ELMA ^a^		Crude
			Days (95% CI) per 2 years		Days per 2 years		Days (95% CI) per 2 years		Days per 2 years		Days (95% CI) per 2 years		Days per 2 years		Days (95% CI) per 2 years		Days per 2 years
**Men**																	
	18–34 years																
		Ref 0 of 3	636.5 (625.9–647.2)		648.7		15.8 (10.3–21.3)		13.4		7.0 (3.3–10.8)		6.9		68.3 (59.3–77.2)		60.9
		1 of 3	-4.3 (-19.4–10.8)		-0.9		-2.0 (-9.7–5.8)		+3.2		+4.7 (-0.6–9.9)		+2.8		+4.0 (-8.7–16.7)		-5.0
		2 of 3	-12.8 (-27.9–2.3)		-15.1		+18.0 (10.3–25.8) ^b^		+12.8		+14.6 (9.4–19.9) ^b^		+11.8		-26.9 (-39.7– -14.2) ^b^		-9.4
		3 of 3	+35.2 (20.0–50.3) ^b^		-2.6		-1.8 (-9.6–5.9)		+7.9		+2.5 (-2.7–7.8)		+13.4		-49.8 (-62.6– -37.1) ^b^		-18.6
	35–49 years																
		Ref 0 of 3	700.8 (696.1–705.5)		703.1		16.2 (12.8–19.6)		14.1		3.7 (2.1–5.2)		3.5		7.7 (5.9–9.6)		9.0
		1 of 3	-7.2 (-13.9–-0.5)		-3.0		+3.2 (-1.6–8.0)		+4.0		+0.6 (-1.6–2.8)		+0.1		+0.0 (-2.5–2.6)		-0.8
		2 of 3	-15.8 (-22.5–-9.1) ^b^		-23.4		+6.8 (1.9–11.6) ^b^		+10.9		+2.2 (0.1–4.4)		+3.3		+2.7 (0.2–5.3)		+9.5
		3 of 3	-46.3 (-53.0–-39.6) ^b^		-38.0		+32.7 (27.9–37.5) ^b^		+21.0		+3.6 (1.5–5.8) ^b^		+4.9		+7.3 (4.7–9.9) ^b^		+8.6
	50–64 years																
		Ref 0 of 3	667.2 (661.6–672.8)		639.9		22.1 (19.4–24.9)		18.2		4.5 (0.7–8.3)		4.3		2.5 (0.6–4.4)		2.5
		1 of 3	-8.1 (-16.0–-0.1)		+0.3		+3.9 (-0.0–7.8)		+5.8		+6.8 (1.4–12.2)		+3.8		+1.1 (-1.6–3.8)		+1.0
		2 of 3	-36.5 (-44.5–-28.6) ^b^		-7.1		+23.7 (19.8–27.6) ^b^		+13.9		+15.7 (10.3–21.1) ^b^		+10.4		+7.8 (5.0–10.5) ^b^		+2.0
		3 of 3	-32.9 (-40.9–-25.0) ^b^		+11.6		+26.0 (22.1–29.9) ^b^		+18.5		+30.2 (24.8–35.6) ^b^		+9.3		+0.2 (-2.6–2.9)		-0.5
**Women**																	
	8–34 years																
		Ref 0 of 3	557.2 (541.1–573.2)		549.4		25.8 (16.8–34.7)		27.1		7.8 (2.6–13.1)		9.6		146.1 (138.1–154.2)		143.7
		1 of 3	-19.7 (-42.3–3.0)		-16.2		+15.7 (3.0–28.4)		+8.9		+3.5 (-3.9–10.9)		+2.2		-1.7 (-13.1–9.7)		+5.2
		2 of 3	-19.5 (-42.2–3.2)		-23.1		+9.6 (-3.1–22.3)		+19.7		+18.0 (10.5–25.4) ^b^		+10.7		-22.3 (-33.7– -10.9) ^b^		-7.2
		3 of 3	-80.5 (-103.1–-57.8) ^b^		-71.8		+46.0 (33.2–58.7) ^b^		+34.6		+15.7 (8.3–23.2) ^b^		+14.8		+1.0 (-10.4–12.4)		+22.6
	35–49 years																
		Ref 0 of 3	672.3 (664.8–679.7)		681.7		31.1 (27.4–34.8)		26.8		6.2 (2.9–9.5)		4.7		16.2 (12.0–20.5)		15.9
		1 of 3	-19.8 (-30.4–-9.3) ^b^		-17.1		+11.6 (6.4–16.9) ^b^		+12.8		+1.7 (-3.0–6.3)		+2.3		+1.7 (-4.3–7.7)		+2.6
		2 of 3	-51.5(-62.1–-41.0) ^b^		-43.6		+25.0 (19.8–30.3) ^b^		+30.0		+11.5 (6.8–16.1) ^b^		+8.9		+21.3 (15.3–27.3) ^b^		+5.5
		3 of 3	-81.6 (-92.2–-71.1) ^b^		-54.6		+50.7 (45.4–55.9) ^b^		+40.8		+33.6 (29.0–38.3) ^b^		+9.3		+6.9 (0.9–12.9)		+5.4
	50–64 years																
		Ref 0 of 3	661.3 (655.9–666.8)		626.8		30.0 (26.4–33.6)		26.2		7.2 (5.6–8.7)		4.6		2.5 (1.3–3.8)		2.7
		1 of 3	-17.0 (-24.7–-9.3) ^b^		-10.4		+15.0 (9.9–20.2) ^b^		+11.1		+3.1 (0.9–5.2) ^b^		+2.7		+1.3 (-0.4–3.1)		+0.6
		2 of 3	-37.9 (-45.6–-30.2) ^b^		-27.3		+32.7 (27.6–37.9) ^b^		+28.0		+7.6 (5.4–9.7) ^b^		+4.7		+1.5 (-0.3–3.3)		+0.8
		3 of 3	-44.9 (-52.5–-37.2) ^b^		-33.0		+40.5 (35.3–45.6) ^b^		+41.7		+9.6 (7.5–11.8) ^b^		+8.5		+3.8 (2.0–5.6) ^b^		+2.6

^a^ ELMA results are adjusted by inverse probability weights on: body mass index, smoking, weekly alcohol consumption, physical activity, disease treatment, state time arrangement, employment sector, highest educational level, and number of survey waves. ^b^ 5% significant.

For example, for women aged 35–49 years, the number of work days decreased by 19.8 days at one indicator, 51.5 days at two indicators, and 81.6 days at all three indicators, with a corresponding increase in sickness absence of 11.6, 25.0, and 50.7 days. For the smallest group of young men, no distinct pattern was seen.

The crude estimates in table 2 generally followed the ELMA results for the reference group but deviate when compared to the employees experiencing work stress.

Supplementary material H presents the ELMA results for the absorbing states of retirement, disability pension, and death. Table H1 shows a postponed retirement (4.2 days to 26.4 days) for older employees having one to three work-stress indicators – most pronounced for the men. Supplementary material I presents the results of the multi-state cox-regressions.

**Figure 2:**
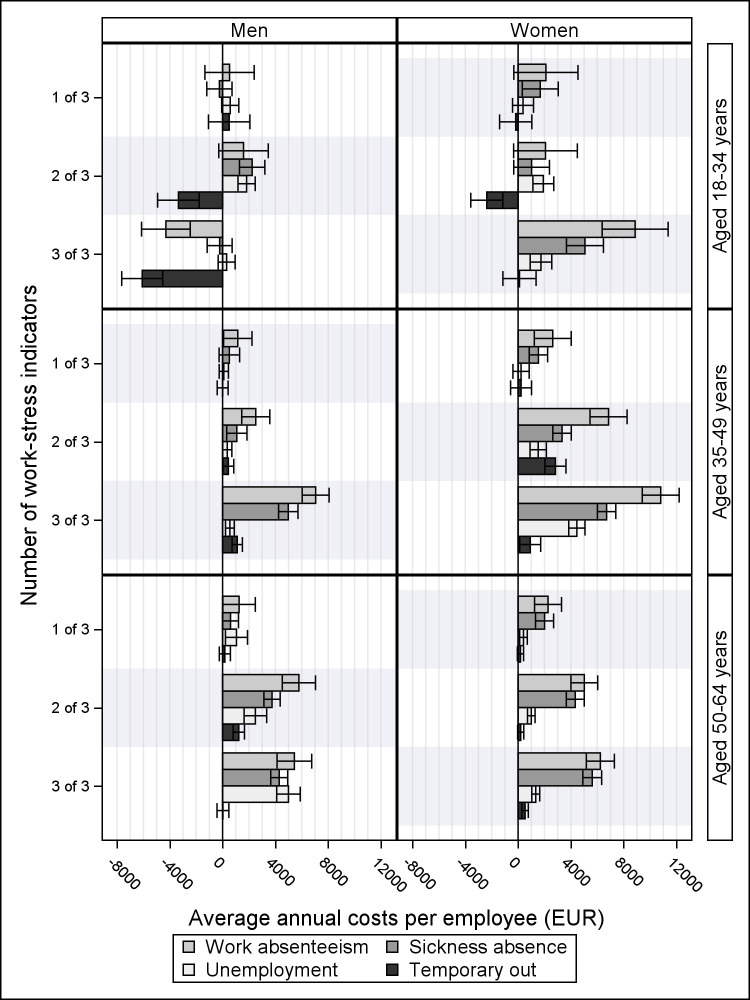
Mean annual costs of work absenteeism per employee at the euro 2022 price level. By the number of work-stress indicators – and the contribution of sickness absence, unemployment, and temporary out. Adjusted by inverse probability weights on body mass index, smoking, weekly alcohol consumption, physical activity, disease treatment, state-time arrangement, employment sector, highest educational level, and number of survey waves.

Figure 2 shows that the costs associated with work stress closely followed the pattern shown for the ELMA results in table 2. The numbers in figure 2 correspond to the cost results shown in table 3 (note that the cost measures in table 3 are annual and not for the two-year follow-up).

**Table 3:** Estimated labor market affiliation (ELMA) results converted to annual standardized (37-hours per week) average costs of work absenteeism per full-time employee from increased work stress levels by sex and age group and the contribution of sickness absence, unemployment, and temporary out. (All priced at EUR 2022 value). [CI=confidence interval].

Number of work-stress indicators			Work absenteeism		Sickness absence		Unemployment		Temporary out
			Average EUR per employee per year (95% CI)		Average EUR per employee per year (95% CI)		Average EUR per employee per year (95% CI)		Average EUR per employee per year (95% CI)
**Men**									
	18–34 years								
		1 of 3	527.2 (516.1–538.2)		-242.0 (-247.7– -236.4)		578.6 (574.8–582.5)		499.0 (489.7–508.2)
		2 of 3	1585.6 (1567.4–1603.8)		2238.8 (2229.4–2248.1)		1818.2 (1811.8–1824.5)		-3346.1 (-3361.5– -3330.8)
		3 of 3	-4294.3 (-4326.3– -4262.4)		-223.4 (-239.8– -207.1)		306.6 (295.5–317.7)		-6089.5 (-6116.4– -6062.6)
	35–49 years								
		1 of 3	1150.9 (1145.7–1156.0)		512.2 (508.5–515.9)		92.1 (90.4–93.8)		4.6 (2.6–6.6)
		2 of 3	2512.4 (2504.2–2520.6)		1078.1 (1072.2–1084.1)		355.0 (352.3–357.7)		436.6 (433.5–439.8)
		3 of 3	7047.6 (7036.0–7059.2)		4975.7 (4967.3–4984.0)		552.6 (548.8–556.4)		1109.3 (1104.8–1113.7)
	50–64 years								
		1 of 3	1250.0 (1243.1–1256.9)		601.1 (597.8–604.5)		1052.3 (1047.6–1057.0)		171.3 (168.9–173.7)
		2 of 3	5780.0 (5768.9–5791.1)		3744.8 (3739.3–3750.2)		2483.6 (2476.1–2491.2)		1228.4 (1224.6–1232.2)
		3 of 3	5437.1 (5418.3–5455.9)		4288.7 (4279.5–4297.9)		4988.2 (4975.4–5000.9)		26.2 (19.8–32.6)
Total ^a^			1903.0 (1892.3–1913.7)		1141.9 (1134.4–1149.4)		842.4 (837.4–847.5)		-43.3 (-50.1–-36.4)
**Women**									
	18–34 years								
		1 of 3	2106.6 (2093.2–2119.9)		1679.8 (1672.3–1687.3)		374.2 (369.8–378.6)		-180.5 (-187.2–-173.7)
		2 of 3	2074.9 (2056.3–2093.5)		1022.7 (1012.3–1033.1)		1910.8 (1904.8–1916.9)		-2370.4 (-2379.8–-2361.1)
		3 of 3	8865.2 (8835.4–8895.0)		5064.5 (5047.8–5081.2)		1733.6 (1723.9–1743.4)		106.2 (91.2–121.2)
	35–49 years								
		1 of 3	2625.7 (2619.7–2631.6)		1539.5 (1536.5–1542.5)		224.0 (221.4–226.6)		225.3 (221.9–228.7)
		2 of 3	6850.8 (6842.2–6859.4)		3329.6 (3325.3–3333.9)		1523.2 (1519.5–1527.0)		2828.1 (2823.2–2832.9)
		3 of 3	10802.6 (10789.2–10815.9)		6704.2 (6697.5–6710.8)		4451.7 (4445.8–4457.5)		917.2 (909.6–924.8)
	50–64 years								
		1 of 3	2268.2 (2263.3–2273.1)		2005.2 (2001.9–2008.5)		408.4 (407.0–409.8)		179.7 (178.6–180.8)
		2 of 3	5015.5 (5008.5–5022.5)		4331.2 (4326.6–4335.9)		999.3 (997.3–1001.3)		197.1 (195.5–198.7)
		3 of 3	6231.0 (6219.5–6242.4)		5616.9 (5609.2–5624.6)		1337.5 (1334.2–1340.7)		528.9 (526.3–531.6)
Total ^a^			3909.0 (3898.1–3919.8)		2613.6 (2606.9–2620.3)		792.0 (787.7–796.3)		274.9 (269.9–279.8)

^a^ The Total estimate uses a standardized weighted average. Adjusted by inverse probability weights on: body mass index, smoking, weekly alcohol consumption, physical activity, disease treatment, state time arrangement, employment sector, highest educational level, and number of survey waves.

Table 3 shows the annual average costs per employee, which was estimated as a weighted average of the sum of the sex- and age-specific estimates. The supplementary material table F1 contains the corresponding weighted number of employees and the total yearly costs. The total weighted sample (N=1 230 754) represents all Danish employees matching the study sample, corresponding to 54% coverage of all full-time employees (N=2 275 785 full-time employees in the Danish labor force in 2022, aged 18–64 years) (34).

The weighted total average annual cost of work absenteeism was €1903 and €3909 for men and women, respectively, per employee with one, two, or three work-stress indicators. Payments of wages to sick-listed employees constituted 60% of the cost of work absenteeism for men and 68% for women. For men, the remaining cost of work absenteeism concerned employees being unemployed, while for women, 7% of the remaining cost was due to time spent in the “temporary out” state concerning maternity leave.

The highest age-divided annual average costs of work absenteeism per employee were for women aged 35–49 years with three indicators of work stress (10 €802.60). Partly originating from increased sickness absence (€6704.20), unemployment (€4451.70), and the temporary out state (€917.20). The lowest annual average costs of work absenteeism were observed among young male employees (-€4294.30). Overall, most of the work absenteeism loss originates from increased sickness absence costs, but increased loss due to employees becoming unemployed and time spent in the temporary out state, were also critical.

The supplementary material table F1 shows the total annual costs for the weighted sample size. The total annual cost of work absenteeism for men was 35% (€305.2 million) of the total annual cost for women (€868.5 million), and the contribution of increased sickness absence was 60% for men (€183.2 million), while it was 67% for women (€580.7 million).

Supplementary table F1 additionally shows that the yearly costs of work absenteeism were generally lower for men than women. Among men, those aged 50-64 with two work-stress indicators had the highest total annual costs of work absenteeism (€74.3 million). This was 59% less than the highest costs of work absenteeism for women.

The highest total age-divided annual costs of work absenteeism were for women aged 35–49 years with two work-stress indicators (€182.9 million). Women aged 50–64 years with two work-stress indicators had the highest contribution of sickness absence to costs of work absenteeism (€90.9 million), and women aged 35–49 years with three work-stress indicators had the highest contribution of unemployment to costs of work absenteeism (€48.6 million).

Supplementary material E contains results from various sensitivity analyses: (i) an alternative descriptive presentation of the sample by a dichotomous version of the combined work-stress indicator, with a comparison of the annual mean hourly wages by sex, age group, and work-stress indicator; (ii) separate analyses of the individual work-stress indicators: Self-perceived stress, Cohen four-item stress, and job strain; and (iii) cost analysis with part-time employees and crude duration estimates.

The descriptive supplementary tables E1 and E2 show no general difference in the sample between the exposed and nonexposed groups. The sensitivity analyses of the single work-stress measurements show that relying only on one type of work-stress measurement is insecure, as it gives mixed results concerning the cost of work absenteeism and sickness absence both across sexes and ages. Including part-time employees in the cost analysis reduces the total annual costs by €0.1 billion. The cost analysis using the crude estimates was generally lower than the ELMA estimates, which was most widespread on the costs of work absenteeism and least widespread on the cost of sickness absence.

The hypothetical reduction potential presented in supplementary material F shows that the costs of work absenteeism and sickness absence are reduced by 7%, >30%, and >60% if the level of work-related stress within the employees is reduced by 10%, 50%, and 100%, respectively.

## Discussion

The aim of the present study was to analyze work-related stress in a large sample of Danish employees to estimate labor market-related costs. We observed substantial economic costs associated with the number of work-stress indicators in terms of self-perceived work stress, the Cohen four-item perceived stress scale, and job strain. The overall average annual cost of work absenteeism per employee was €1903 and €3909 for men and women, respectively. This corresponds to 3.3% of men’s average annual wages and 9.0% of women’s average annual wages (35). We observed significantly higher costs for women than men, and across age ranges, we observed higher costs for middle- to high-aged employees than for young employees. For young male employees with a high level of work stress, we observed both negative and positive associations with labor market costs, reminding us that some work-related stress may incline increased productivity.

The total annual cost of work absenteeism associated with work-related stress was €1.2 billion or 0.3% of the Danish GDP in 2022 (36), of which 67% (€0.8 billion) was for sick-listed employees. The total annual costs of work absenteeism and sickness absence were reduced by €0.1 billion when adjusting for part-time employees. Employees with three work-stress indicators were generally the costliest concerning the value of work absenteeism. However, the yearly costs of work absenteeism depended highly on the occurrence of work stress within the age-sex subgroups and whether a clear pattern of labor market affiliation was evident or not evident, as was the case for young male employees.

A hypothetical analysis of reducing the work-stress level within the employees did show a marked potential for reducing costs of work absenteeism and sickness absence at all three steps: moderately (10%), across widespread (50%), to heavy (100%). Moreover, an analysis comparing the ELMA method with a conventional crude method for estimation of costs of work absenteeism suggested an extensive underestimation of the cost of work-related stress when using the crude measurements.

We did not find any major differences in variable composition between the group of employees with work stress and the reference group. This may have changed if additional explanatory factors were included in the study. However, the combination of lifestyle, health, employment type, and educational factors suggests a strong explanatory basis of variables.

### Comparison with previous studies

We did not find many comparable Danish studies. Juel et al (37) estimated the total cost of work-related stress in 2005 to be approximately €2.0 billion per year, corresponding to €2.9 billion in 2022 (32). The study included costs of sickness absence, early death, and health service expenses and was based on 2000 survey data. Work-related stress was measured solely by job strain. In comparison, the top-down estimate of the total costs of the present study (presented in supplementary material F) was €0.5 billion lower. It is, however, difficult to make a direct comparison since the two studies do not include the same costs and take different approaches to measuring work-related stress.

Making a direct comparison with studies from countries other than Denmark involves issues that should be considered, for example, unequal access to reliable data, differences in wages, health service expenses, and differences in the composition of the labor force. The analysis design and estimation method may also differ. For example, the present study used the combination of three work-stress indicators: self-perceived stress, the Cohen 4-level scale, and job strain, while most of the studies included in the comprehensive review by Hassard et al (14) solely used job strain as the primary measure of work stress. Several of the studies included by Hassard et al (14) reported substantial expenses linked to work stress despite differences in both study designs and the prevalence of work stress, ranging from 2% to 27%. We observed a comparable prevalence of work stress (13–17%) despite using a slightly more restrictive job strain version. However, our sensitivity analysis on the three single work stress measurements did show mixed results on work absenteeism and sickness absence, suggesting uncertainty regarding the prevalence of work stress when using the measurements separately.

Despite the unique analytical approach and study design, we believe our results are comparable to other European work-stress studies since many European employees can receive wages during sickness absence, such as in the UK, The Netherlands, and Scandinavian countries. Moreover, by appropriately adjusting the overall estimated annual costs, the results may become comparable for hypothetical effect comparison with foreign interventions and policy-making (38).

### Strengths and limitations

The study has several strengths. First, by including four waves of WEHD survey data, we built a large sample size spanning a long period, increasing the analysis strength by incorporating both single and repeated measurements. The linkage to multiple longitudinal registers with date-based records on wage payments and social benefits is a profound strength, especially when the analysis preserves the dynamic of the individual labor transitions in terms of the multi-state modeling and the ELMA method. An additional strength concerns the multiple-angle detection of work-related stress through three acknowledged work-stress indicators.

There are also some limitations to the study. First, the sample population does not include small companies with <10 employees due to a lack of information on short-term sickness absence in the registers. Small companies constitute a large part of the Danish labor market. Second, only a few individuals entered the states of “disability pension” and “death” despite work-related stress, which may be related to these outcomes. This was likely due to the relatively short follow-up period and the age limit of 64 years. Third, the results cannot be used for individual predictions of expected costs of work absenteeism for a specific employee who experiences work stress. Instead, the results are of a general character expressing the mean expected costs of work absenteeism for groups of employees exposed to work stress. Fourth, the study included both part- and full-time benefits, as well as part- and full-time wage payments. If multiple payments were recorded simultaneously, then we prioritized between the payments made. This prioritization likely resulted in slightly underestimated durations of working time and overestimated durations of the other states. Fifth, any exposure to private life-related stress was likely to interfere and may trigger stress at work, and the individual contribution of different sources of stress types may be difficult to separate. Additionally, the reference group contains employees reporting a high level of personal-related stress. Sixth, the lack of individual-based objective information on medication and disease may have caused bias if, eg, the use of certain medications was more frequent within the exposed group. This may, for example, cause an underestimation of the work-stress cost if the exposed employees more frequently used pain medication to reduce headaches, thereby reducing the risk of sickness absence. Seventh, the restricted two-year follow-up period favors short-term consequences of work stress and was likely to underrate long-term consequences such as continuous sickness absence. Moreover, the study concerns a period with a fairly constant prevalence of work stress of 28–29%, which may restrict the results to 2012–2020. Eighth, the cost analysis primarily estimated the work-stress-related costs of work absenteeism. However, we expect work stress to have other costs, eg, concerning specific healthcare services and likewise costs related to the individual quality of life. Therefore, obtaining a more solid estimate of the total employer and employee costs of work stress will require more research.

### Concluding remarks

We showed that work-related stress was associated with substantial labor market costs. This study estimated that the total annual value of work absenteeism of work-related stress in Denmark was €305.2 million for men and €868.5 million for women, or 0.3% of the GDP. The long-term and social health costs of work-related stress are likely even higher, depending on the possibility of quantifying every aspect of the problem. However, given this already sizeable economic burden, the prevention of work-related stress is a major occupational health concern, and the development of effective interventions to achieve this aim should be given high priority.

### Role of the funding source

The Danish National Research Centre for the Working Environment supported this study. The funder of the study had no role in the study design, data collection, data analysis, data interpretation, or writing of the report. The corresponding author had full access to all the data and had the final responsibility to submit it for publication.

### Ethics approval

According to Danish law, research studies that use solely survey and register data do not need approval from the National Committee on Health Research Ethics (Den Nationale Videnskabetiske Komité).

## Supplementary material

Supplementary material

## Data Availability

Data are available in the Researcher access portal at the Statistics Denmark website: www.dst.dk/en/TilSalg/Forskningsservice.

## References

[1] Götz S, Hoven H, Müller A, Dragano N, Wahrendorf M. Age differences in the association between stressful work and sickness absence among full-time employed workers: evidence from the German socio-economic panel. Int Arch Occup Environ Health 2018 May;91(4):479–96. 10.1007/s00420-018-1298-3 29487994 PMC5908813

[2] Mortensen J, Dich N, Lange T, Alexanderson K, Goldberg M, Head J et al. Job strain and informal caregiving as predictors of long-term sickness absence: A longitudinal multi-cohort study. Scand J Work Environ Health 2017 Jan;43(1):5–14. 10.5271/sjweh.3587 27556905

[3] Thorsen SV, Pedersen J, Flyvholm MA, Kristiansen J, Rugulies R, Bültmann U. Perceived stress and sickness absence: a prospective study of 17,795 employees in Denmark. Int Arch Occup Environ Health 2019 Aug;92(6):821–8. 10.1007/s00420-019-01420-9 30810815 PMC6609587

[4] Nieuwenhuijsen K, Bruinvels D, Frings-Dresen M. Psychosocial work environment and stress-related disorders, a systematic review. Occup Med (Lond) 2010 Jun;60(4):277–86. 10.1093/occmed/kqq081 20511268

[5] Holmgren K, Fjällström-Lundgren M, Hensing G. Early identification of work-related stress predicted sickness absence in employed women with musculoskeletal or mental disorders: a prospective, longitudinal study in a primary health care setting. Disabil Rehabil 2013 Mar;35(5):418–26. 10.3109/09638288.2012.695854 22804618

[6] Theorell T, Hammarström A, Gustafsson PE, Magnusson Hanson L, Janlert U, Westerlund H. Job strain and depressive symptoms in men and women: a prospective study of the working population in Sweden. J Epidemiol Community Health 2014 Jan;68(1):78–82. 10.1136/jech-2012-202294 24052515

[7] Mather L, Bergström G, Blom V, Svedberg P. High Job Demands, Job Strain, and Iso-Strain are Risk Factors for Sick Leave due to Mental Disorders: A Prospective Swedish Twin Study With a 5-Year Follow-Up. J Occup Environ Med 2015 Aug;57(8):858–65. 10.1097/JOM.0000000000000504 26247639

[8] Pedersen J, Solovieva S, Thorsen SV, Andersen MF, Bültmann U. Expected Labor Market Affiliation: A New Method Illustrated by Estimating the Impact of Perceived Stress on Time in Work, Sickness Absence and Unemployment of 37,605 Danish Employees. Int J Environ Res Public Health 2021 May;18(9):4980. 10.3390/ijerph18094980 34067104 PMC8124718

[9] Hintsa T, Kouvonen A, McCann M, Jokela M, Elovainio M, Demakakos P. Higher effort-reward imbalance and lower job control predict exit from the labour market at the age of 61 years or younger: evidence from the English Longitudinal Study of Ageing. J Epidemiol Community Health 2015 Jun;69(6):543–9. 10.1136/jech-2014-205148 25631860 PMC4453492

[10] Juvani A, Oksanen T, Salo P, Virtanen M, Kivimäki M, Pentti J et al. Effort-reward imbalance as a risk factor for disability pension: the Finnish Public Sector Study. Scand J Work Environ Health 2014 May;40(3):266–77. 10.5271/sjweh.3402 24247977

[11] Mäcken J. Work stress among older employees in Germany: effects on health and retirement age. PLoS One 2019 Feb;14(2):e0211487. 10.1371/journal.pone.0211487 30716089 PMC6361437

[12] Pedersen J, Bjorner JB, Burr H, Christensen KB. Transitions between sickness absence, work, unemployment, and disability in Denmark 2004-2008. Scand J Work Environ Health 2012 Nov;38(6):516–26. 10.5271/sjweh.3293 22441355

[13] Pedersen J, Framke E, Thorsen SV, Sørensen K, Andersen MF, Rugulies R et al. The linkage of depressive and anxiety disorders with the expected labor market affiliation (ELMA): a longitudinal multi-state study of Danish employees. Int Arch Occup Environ Health 2023 Jan;96(1):93–104. 10.1007/s00420-022-01906-z 35857111 PMC9823083

[14] Hassard J, Teoh KR, Visockaite G, Dewe P, Cox T. The cost of work-related stress to society: A systematic review. J Occup Health Psychol 2018 Jan;23(1):1–17. 10.1037/ocp0000069 28358567

[15] exchangerates.org.uk. Exchange Rates UK [Accessed 2023 12-9]. Available from: https://www.exchangerates.org.uk/USD-EUR-spot-exchange-rates-history-2014.html

[16] Safe Work Australia. The Cost of Work-related Injury and Illness for Australian Employers, Workers and the Community: 2008–09 [Accessed 2022 15-6]. Available from: https://www.safeworkaustralia.gov.au/system/files/documents/1702/cost_of_work-related_injury_and_disease.pdf

[17] State Secretariat for Economic Affairs (SECO). Ramaciotti D, Perriard J. Die Kosten von Stress in der Schweiz [English translation.] Accessed 2022 17-08. Available from: https://www.seco.admin.ch/seco/de/home/Publikationen_Dienstleistungen/Publikationen_und_Formulare/Arbeit/Arbeitsbedingungen/Studien_und_Berichte/die-kosten-des-stresses-in-der-schweiz.html

[18] The Sainsbury Centre for mental Health. Mental Health at Work: Developing the business case, Policy Paper 8 [Accessed 2022 17-08]. Available from: https://www.centReformentalhealth.org.uk/publications/mental-health-work-developing-business-case

[19] Sculpher MJ, McGuire MD. The role and estimation of productivity costs in economic evaluation. Economic evaluation in health care: Oxford University Press; 2001. p. 94–112.

[20] Pedersen J, Bjorner JB, Andersen LL. Physical work demands and expected labor market affiliation (ELMA): prospective cohort with register-follow-up among 46 169 employees. Scand J Work Environ Health 2022 Nov;48(8):641–50. 10.5271/sjweh.4050 35789276 PMC10546615

[21] Cohen S, Kamarck T, Mermelstein R. A global measure of perceived stress. J Health Soc Behav 1983 Dec;24(4):385–96. 10.2307/2136404 6668417

[22] Schnall PL, Landsbergis PA, Baker D. Job strain and cardiovascular disease. Annu Rev Public Health 1994;15:381–411. 10.1146/annurev.pu.15.050194.002121 8054091

[23] Niedhammer I, Bertrais S, Witt K. Psychosocial work exposures and health outcomes: a meta-review of 72 literature reviews with meta-analysis. Scand J Work Environ Health 2021 Oct;47(7):489–508. 10.5271/sjweh.3968 34042163 PMC8504166

[24] Johnsen NF, Thomsen BL, Hansen JV, Christensen BS, Rugulies R, Schlünssen V. Job type and other socio-demographic factors associated with participation in a national, cross-sectional study of Danish employees. BMJ Open 2019 Aug;9(8):e027056. 10.1136/bmjopen-2018-027056 31427315 PMC6701570

[25] CPR-Administration. Can I get a civil registration number? [Accessed 2021 28-9]. Available from: https://cpr.dk/english/moving-to-denmark/

[26] Statistics Denmark. Periods of Absence register [Accessed 2022 14-6]. Available from: https://www.dst.dk/da/Statistik/dokumentation/Times/fravaer

[27] de Wreede LC, Fiocco M, Putter H. The mstate package for estimation and prediction in non- and semi-parametric multi-state and competing risks models. Comput Methods Programs Biomed 2010 Sep;99(3):261–74. 10.1016/j.cmpb.2010.01.001 20227129

[28] Pedersen J, Bjorner JB. Worklife expectancy in a cohort of Danish employees aged 55-65 years - comparing a multi-state Cox proportional hazard approach with conventional multi-state life tables. BMC Public Health 2017 Nov;17(1):879. 10.1186/s12889-017-4890-7 29141598 PMC5688741

[29] Pedersen J, Schultz BB, Madsen IE, Solovieva S, Andersen LL. High physical work demands and working life expectancy in Denmark. Occup Environ Med 2020 Aug;77(8):576–82. 10.1136/oemed-2019-106359 32398291 PMC7402449

[30] Pedersen J, Thorsen SV, Andersen MF, Hanvold TN, Schlünssen V, Bültmann U. Impact of depressive symptoms on worklife expectancy: a longitudinal study on Danish employees. Occup Environ Med 2019 Nov;76(11):838–44. 10.1136/oemed-2019-105961 31582420 PMC6839798

[31] Pedersen J, Bjorner JB, Andersen LL. Physical work demands and expected labor market affiliation (ELMA): prospective cohort with register-follow-up among 46 169 employees. Scand J Work Environ Health 2022 Nov;48(8):641–50. 10.5271/sjweh.4050 35789276 PMC10546615

[32] Statistics Denmark. Wage index [Accessed 2023 06-03]. Available from: www.statistikbanken.dk/LONS60

[33] Evers S, Goossens M, de Vet H, van Tulder M, Ament A. Criteria list for assessment of methodological quality of economic evaluations: Consensus on Health Economic Criteria. Int J Technol Assess Health Care 2005;21(2):240–5. 10.1017/S0266462305050324 15921065

[34] Statistics Denmark. Number of Danish full-time employees 2022 [Accessed 2023 11-05]. Available from: https://www.statistikbanken.dk/RAS204

[35] Euroinvestor. Kvinder får stadig ikke lige så høj løn som mænd – forskellen er den største i 16 år [English translation]. Accessed 2023 20-9. Available from: https://www.euroinvestor.dk/privatoekonomi/kvinder-faar-stadig-ikke-lige-saa-hoej-loen-som-maend-forskellen

[36] Statistics Denmark. GDP 2022 [Accessed 2023 20-9]. Available from: https://www.dst.dk/en/Statistik/emner/oekonomi/nationalregnskab/noegletal-for-nationalregnskabet-bnp

[37] Juel K, Sørensen J, Brønnum-Hansen H. Risikofaktorer og folkesundhed i Danmark. 2006. [English translation] Available from: https://www.sdu.dk/da/sif/rapporter/2006/risikofaktorer_og_folkesundhed_i_danmark

[38] O’Keefe LC, Brown KC, Christian BJ. Policy perspectives on occupational stress. Workplace Health Saf 2014 Oct;62(10):432–8. 10.3928/21650799-20140813-02 25139784

