# Supplementary material for: The labor market costs of work-related stress: A longitudinal study of 52 763 Danish employees using multi-state modeling
Source: Scand J Work Environ Health. 2024 Feb 28;50(2):61–72. doi: 10.5271/sjweh.4131 (PMC10927069; doi:10.5271/sjweh.4131)
Supplement: Supplementary material [file SJWEH-50-61-S001.pdf]

The labor market costs of work-related stress: A longitudinal study of 52 763 Danish employees using multi-state modeling[1]

by Jacob Pedersen, PhD,[2] Brian Krogh Graversen, PhD, Kristian Schultz Hansen, Professor, Ida Elisabeth Huitfeldt Madsen, PhD

1. Supplementary material
2. Correspondence to: Jacob Pedersen, PhD, National Research Centre for the Working Environment, Lersø Parkallé 105, DK-2100 Copenhagen Ø, Denmark. [E-Mail: jpe@nfa.dk]

A - Details on the WEHD survey data

The four waves of the WEHD survey contain a mix of individuals from stratified workplace samples and random samples. To be included in the survey individuals have to be 18-64 years old, be employed for at least 35 hours per month, and earn at least 3000 Danish Kroner (DKK) per month (~EUR432) (1) in up to six months before the survey was sent to the invited participants.

In 2012 the survey was sent to a random sample of 35 039 individuals, and to 15 767 individuals who were randomly selected from five specially selected types of industries; 1) knowledge, 2) private service, 3) personal caretaking, 4) industry, and 5) construction. The industry sample was stratified by company size in terms of the number of annual full-time workers (10-34, 35-99, 100-249, 250-499, and 500+), and by the five industry types each including 408 companies. This was done to secure a sufficient number of individuals within the selected industry types and companies. The response dates of the 2012 wave go from 12th April 2012 to 16th September 2012, and the final response rate was 51.5%.

In 2014, 16 138 respondents from 2012 aged lower than 65 years, were re-invited to participate, except for the individuals from the selected industry sample. Moreover, a new random sample consisting of 35 023 individuals was invited to participate. The response dates go from 19th March. 2014 to 15th August 2014, and the final response rate was 57.3%.

In 2016, all 15 234 respondents from 2012 aged lower than 65 years, were re-invited to participate, except for the individuals from the selected industry samples. A new industry sample consisting of 15 507 individuals was made using the same selection criteria as the industry sample in the 2012 wave. Moreover, a new random sample of 35 000 individuals aged 18-64 years was invited to participate. The response dates go from 9th March. 2016 to 4th September 2016, and the final response rate was 52.9%.

In 2018, all 13 064 respondents from 2012 and all 14 036 respondents from 2016 aged lower than 65 years, were re-invited to participate, except for the individuals from the selected industry samples. Moreover, a

new random sample of 36 634 individuals aged 18-64 years was invited to participate. The response dates go from 18th December 2017 to 17th July 2018, and the final response rate was 59.1%.

First, the participants were sent a letter with a link to an online version of the questionnaire. Those who did not react to the first letter received another letter. If still no reaction, then the invited employees were contacted by phone or SMS by an analysis agency that encouraged them to participate by sending them a questionnaire. Finally, a paper version with a stamped reply envelope was sent to invited employees who still had not responded.

The provided individual sample weights for making the sample of respondents representable for all Danish employees are based on the following characteristics; sex, age, occupation, industry type, and industry size.

Source: at.dk/media/5994/danskernes-arbejdsmiljoe-2016.pdf and NFA documentation on WEHD

B – Description of the sample selection process and flow chart

Accompanying the RoWA register is individual weights for all private employees. The weights are based on the sampling probability for employees in the private sector. The weights for public employees are equal to the value one. We excluded records that could not be linked to a RoWA weight causing a 31% reduction in follow-up periods (39 108) and a 30% reduction in unique respondents (N=25 364).

To confirm the individual labor market attachment at the survey date, we excluded follow-up periods for individuals who did not have a minimum of 14 work days - counted up to eight weeks before the answering date (excluding 6249 follow-up periods, N=4977 respondents). We did this, since the time between the WEHD sample selection and the individual answering date can be up to six months.

Then we excluded periods starting in the retirement or the disability pension state (614 follow-up periods, N=80 respondents). We also removed follow-up periods if the respondent was not in the age range between 18 and 64 years at the start of the follow-up (295 follow-up periods, N=137 respondents). Since we identified the employer-id at the survey answering date, we finally censored any follow-up time if a new employer-id was registered - including periods of which the first part of the follow-up was truncated due to missing weights (3056 follow-up periods, N=1950 respondents).

The final sample of N=52 763 unique respondents, was divided into six subsamples according to sex and age at the start of follow-up (18-34 years, 35-49 years, and 50-64 years). Of the respondents; 52% answered only one of the four waves of questionnaires, 24% two waves of questionnaires, 10% three waves of questionnaires, and 14% answered all four questionnaire waves – summing to 75 537 follow-up periods.

Figure 1B - Flow-chart

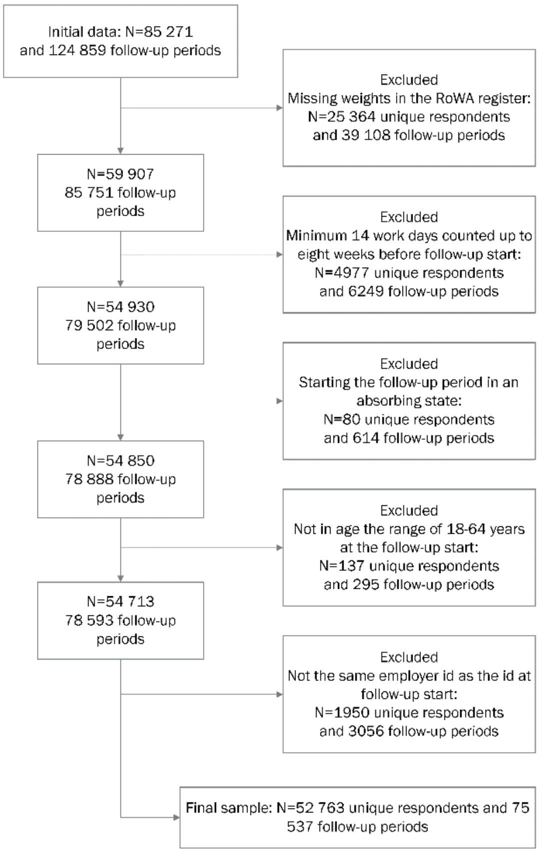

C - Details on the three work-stress indicators

(i) Self-perceived stress was measured with two questions in the WEHD 2012- 2018 surveys; 1) "How often have you felt stressed in the last two weeks?" (Response: always, often, sometimes, seldom, or never) 2) "What was the most important source of your stress?" (Response: work, personal life, or work and personal life). Individuals who responded "always" or "often" to the first item and "work stress", or "work and personal stress" to the second item were categorized as having work-related stress. Those responding "personal stress" and the rest were categorized as not having work-related stress.

(ii) The four-item perceived stress scale by Cohen, which was slightly modified to specifically measure work-related stress; "In the last month, how often have you …" 1) "felt that you were unable to control the important things in your life?" 2) "felt difficulties at work were piling up so high that you could not overcome them?" 3) "felt confident about your ability to handle problems at work?" 4) "felt that things at work were going your way?" (Response: always, often, sometimes, seldom, and never). The individual

response to the first two items was scored four to zero, and the response to the last two items was scored zero to four. Individuals were categorized as having work-related stress if the sum of the four-item scores was eight or more, with the sum of scores ranging from zero to sixteen.

(iii) Job strain was measured using a sex-specific cut-off, made from five items on quantitative demands; "How often …" 1) "do you find that you have enough time for your work tasks?" 2) "how often do you have deadlines that are hard to meet? 3) "do you get unexpected work tasks, that put you under time pressure?" 4) "do you make yourself available outside the normal working hours?" 5) "is it necessary to work overtime?" (Response: always, often, sometimes, seldom, never). Two items on influence/job control; "How often do you have an influence on …" 1) "how you solve your work tasks?" 2) "when you solve your work tasks?" (Response: always, often, sometimes, seldom, never). The first item concerning quantitative demand scored five to one, while the remaining items concerning quantitative demand and the two items concerning job control scored one to five.

For job-strain, the most common procedure for finding the exposed employees is by comparing the individual score on quantitative demands and influence with the sample median score of the quantitative demands and the median score of influence. This procedure typically marks more employees as having stress than the 4-item Cohen and the self-perceived stress questions. For this reason, we made sensitivity and specificity analyses on each of the three stress indicators against the presence of major depression in terms of an MDI score of 21 and above. From the sensitivity and specificity analysis, we found the number of false positives very high when using the median job-strain approach. Therefore, we instead used the following method presented by Karasek et al. 2007 (2) for identifying employees with work-related stress; Having a lower mean score on quantitative demands than the population mean and having a higher job control mean score plus one standard deviation than the population median. And/or having a lower mean score minus one standard deviation on quantitative demands than the population mean, and a higher job control mean score than the population median. The identification was made by sex. The remaining individuals were categorized as non-stressed.

D - Detailed description of the states of the labor market model including jurisdictional context and notes on the Danish labor market

Absorbing states: The three absorbing states are defined as states where no further transitions are possible after the first appearance: (i) Death. (ii) Retirement, due to receiving age retirement pension or the voluntary retirement pension. (iii) Disability pension, when receiving full or gradual disability retirement pension due to personal disability.

Recurrent states: The four recurrent states are defined as states where multiple individual periods of the same state are possible during follow-up: (iv) Work (receiving wage payments). (v) Sickness absence (registered as sick-listed by the employer or receiving sickness absence benefit). (vi) Unemployment (receiving social benefits related to unemployment). (vii) Temporary out (other states with the possibility of returning e.g. periods of maternity leave, emigration, no wage or social benefit payments, and of education). The work, sickness absence, and unemployment states constitute the primary states.

Jurisdictional context and notes on the Danish labor market: The Danish labor market is characterized as a flexicurity system with generally high labor market participation rates (73% for females and 77% for males), and low formal employment protection inflicting a high turnover of the workforce. The "security" of the flexicurity contains generous and accessible social benefits e.g. sickness absence, unemployment, and disability pension. Sickness absence benefits are typically paid from 30 days of continuous absence as compensation to the employer for continuing paying salary during the sickness absence period. Two types of unemployment benefits exist - one based on an insurance scheme and one that is accessible to all. Disability pension is available to all with a major disability that limits their workability. The analysis does not distinguish between the disability pension scheme and the gradual disability scheme named flex-job.

The sickness absence benefit is compensation given to the employer for continuing to pay wages to a sick-listed employee. The compensation payment is possible after 30 days of continuous sick listing and will cover a maximum of 90% of the wage with an upper limit of EUR 16.22 (120.68 DKK) per hour, for a standard 37-hour work week. If the sick-listed is unemployed, then the sick-listed person receives the benefit directly from the municipality.

Approximately 80% of the Danish labor force aged 25-59 years are entitled to insurance-based unemployment benefits (3). This means that if unemployed then the person is entitled to receive compensation until 90% of the former salary with a maximum of EUR 19.61 (145.88 DKK) per hour, for a standard 37-hour workweek. The non-insurance-based unemployment benefit is for individuals without sufficient income to support themselves. The benefit payment is lower than the insurance-based scheme and may become even lower if personal savings exist and/or the possibility of economic support from a spouse.

For a person to receive a disability pension, the health-determined workability must be reduced permanently, and there can be no chance of the individual becoming self-supported through income-given-work including other additional social benefits. Before the disability pension application is evaluated by the municipality board, the reduction of the workability must be firmly proven through several social workability programs. Moreover, a disability pension is seldom given to individuals aged below 40 years. If

a person is permanently disabled but still capable of doing some reduced work, the person may receive a flex-job- The flex-job is employment on special terms e.g. that the employer can receive sickness absence benefit compensation from the first day of sick-listing. The right to flex-job is continuously evaluated every 4.5 years for individuals aged below 40 years, this means that the scheme can be stopped.

The official retirement age in Denmark is rising but was 65-66 years during the study follow-up period. It is possible to retire at an earlier age by using a scheme or savings. The most common early retirement method is the Voluntary Early Retirement scheme, which is accessible to employees who registered for the scheme early on since one must have contributed continuously for a minimum of 30 years to the scheme to be able to use it. The Voluntary Early Retirement scheme makes it possible to retire up to five years ahead of the official retirement age.

E – Sensitivity analysis

Table E1 compares the employees with and without work stress indicators. The table distinguishes between employees without work stress (no) and employees with one, two, or three indicators (yes). Table E1 shows that the share of employees with and without work-stress indicators are highly comparable within the variables. The comparability is slightly off for disease treatment, as the number of employees with work-stress indicators is 4-6 percent points higher compared to employees without work-stress indicators. The share of individuals with one, two, or three work-stress indicators in the WEHD 2012 survey was 28%, 29% in both 2014 and 2016, and 28% again in the 2018 survey, indicating a stable frequency of work-stress within the follow-up period.

Table E1 – Descriptive presentation of the study sample at survey start, grouped by: variables, sex, and dichotom identification of work-stress indicators (no/yes).

Variables		Men		Women	
		Work-stress indicator			
		No	Yes	No	Yes
		N (%)	N (%)	N (%)	N (%)
Total		15904 (72)	6216 (28)	20458 (67)	10185 (33)
Self-perceived stress	No	15904 (100)	3815 (61)	20458 (100)	5358 (53)
	Yes	. (.)	2401 (39)	. (.)	4827 (47)
Cohen four-item stress	No	15904 (100)	2600 (42)	20458 (100)	4083 (40)
	Yes	. (.)	3616 (58)	. (.)	6102 (60)
Job Strain	No	15904 (100)	3209 (52)	20458 (100)	5486 (54)
	Yes	. (.)	3007 (48)	. (.)	4699 (46)
Body mass index	Underweight	40 (0)	16 (0)	320 (2)	208 (2)
	Normal weight	6141 (39)	2361 (38)	11078 (54)	5407 (53)
	Overweight	6599 (41)	2660 (43)	5203 (25)	2765 (27)
	Obese	2232 (14)	1025 (16)	2616 (13)	1537 (15)

	Not available	892 (6)	154 (2)	1241 (6)	268 (3)
Smoking	Non-smoker	12085 (76)	4763 (77)	16037 (78)	7967 (78)
	Smoker	2972 (19)	1337 (22)	3392 (17)	2080 (20)
	Not available	847 (5)	116 (2)	1029 (5)	138 (1)
Weekly alcohol consumption	None	2005 (13)	952 (15)	4423 (22)	2560 (25)
	Moderate	5457 (34)	2171 (35)	8968 (44)	4378 (43)
	High	7596 (48)	2979 (48)	6027 (29)	3104 (30)
	Not available	846 (5)	114 (2)	1040 (5)	143 (1)
Physical activity	No	8851 (56)	3618 (58)	12144 (59)	6354 (62)
	Yes	7053 (44)	2598 (42)	8314 (41)	3831 (38)
Disease treatment	No	5711 (36)	2031 (33)	7276 (36)	3069 (30)
	Yes	1992 (13)	1027 (17)	3098 (15)	2126 (21)
	Not available	8201 (52)	3158 (51)	10084 (49)	4990 (49)
State time arrangement	95+ pct. of 37 hours/week	13572 (85)	5317 (86)	13436 (66)	6748 (66)
	65-94 pct. of 37 hours/week	2060 (13)	792 (13)	6449 (32)	3170 (31)
	0-64 pct. of 37 hours/week	272 (2)	107 (2)	573 (3)	267 (3)
Employment sector	Private	10251 (64)	3949 (64)	5966 (29)	2943 (29)
	Public	5653 (36)	2267 (36)	14492 (71)	7242 (71)
Highest educational level	Low	1967 (12)	827 (13)	1849 (9)	864 (8)
	Middle	6910 (43)	2514 (40)	7685 (38)	3751 (37)
	High	6927 (44)	2827 (45)	10854 (53)	5523 (54)
	Not available	100 (1)	48 (1)	70 (0)	47 (0)
Number of Survey waves	1 of 4	11774 (74)	4639 (75)	14519 (71)	7347 (72)
	2 of 4	2622 (16)	1004 (16)	3703 (18)	1740 (17)
	3 of 4	716 (5)	276 (4)	1012 (5)	536 (5)
	4 of 4	792 (5)	297 (5)	1224 (6)	562 (6)

Table E2 shows the average hourly wage by sex, age group, and number of work-stress indicators. The table shows no difference between wages on age groups and sex, except for women aged 50-64 years with all three work-stress indicators - in which the average hourly wage is significantly higher than the reference group. Overall, the hourly wages are higher for the men than for the women in between age groups. Moreover, it shows that the youngest age group has the lowest average hourly wages.

Table E2 – Annual average hourly wages in EUR by number of work-stress indicators, age group, and sex. All in 2022 price levels.

		Men	Women
Age	Number of work-stress indicators	Mean hourly wage in EUR (95% CI)	Mean hourly wage in EUR (95% CI)
18-34 years			
	0 of 3	33.4 (32.8 - 34.0)	29.5 (29.1 - 29.9)
	1 of 3	33.7 (32.7 - 34.7)	29.4 (28.8 - 30.0)
	2 of 3	33.8 (32.3 - 35.3)	29.4 (28.4 - 30.4)
	3 of 3	33.8 (30.7 - 37.0)	30.8 (29.1 - 32.4)

35-49 years		
0 of 3	42.8 (42.2 - 43.3)	35.8 (35.5 - 36.1)
1 of 3	43.6 (42.5 - 44.7)	36.1 (35.5 - 36.7)
2 of 3	42.5 (41.0 - 43.9)	36.4 (35.5 - 37.3)
3 of 3	42.1 (39.7 - 44.4)	35.9 (34.6 - 37.2)
50-64 years		
0 of 3	42.2 (41.7 - 42.7)	34.9 (34.6 - 35.2)
1 of 3	41.0 (40.0 - 41.9)	35.7 (35.2 - 36.3)
2 of 3	42.2 (40.6 - 43.7)	35.3 (34.6 - 35.9)
3 of 3	43.6 (41.4 - 45.8)	37.0 (35.7 - 38.3)

CI: Confidence Interval.

Sensitivity analysis on the individual work stress indicators

Table E3 shows the average annual cost per employee from an ELMA analysis with self-perceived stress as the explaining variable while including all weights. Compared to the results of Table 3, the total average cost of work absenteeism is markedly reduced below zero for men and increased by 72% for women. The total average cost of sickness absence is increased by approx. 200% for men and decreased by 70% for women.

Table E3 - Annual standardized average costs in EUR of work absenteeism per employee with self-perceived work-stress by sex and age group when compared to employees without self-perceived stress – including the contribution of sickness absence, unemployment, and temporary out. All in 2022 price level.

Self-perceived work-stress	Work absenteeism	Sickness absence	Unemployment	Temporary out
	Average EUR per employee per year (95% CI)	Average EUR per employee per year (95% CI)	Average EUR per employee per year (95% CI)	Average EUR per employee per year (95% CI)
Men				
18-34 years	-8700.4 (-8879.0 - -8521.9)	1861.4 (1832.7 - 1890.2)	1555.6 (1529.2 - 1582.0)	-5810.1 (-5869.8 - -5750.4)
35-49 years	1693.2 (1625.0 - 1761.4)	4746.2 (4732.5 - 4759.9)	-1742.4 (-1754.1 - -1730.6)	1828.6 (1814.9 - 1842.3)
50-64 years	2554.5 (2435.7 - 2673.2)	2681.7 (2649.6 - 2713.9)	2021.3 (2001.4 - 2041.1)	386.5 (380.4 - 392.7)
Total	-627.9 (-702.8 - -553.1)	3383.3 (3366.4 - 3400.2)	253.6 (236.1 - 271.0)	-524.7 (-554.1 - -495.3)
Women				
18-34 years	5456.0 (5364.2 - 5547.7)	-1144.6 (-1176.2 - -1113.1)	268.4 (250.3 - 286.5)	-1601.4 (-1701.3 - -1501.4)
35-49 years	7404.5 (7350.6 - 7458.3)	-2546.8 (-2565.6 - -2528.1)	1217.8 (1208.5 - 1227.1)	-1237.8 (-1247.4 - -1228.2)
50-64 years	6777.7 (6723.3 - 6832.2)	6437.6 (6416.7 - 6458.4)	1345.3 (1341.4 - 1349.2)	476.6 (472.7 - 480.6)
Total	6710.7 (6673.5 - 6747.8)	797.7 (770.0 - 825.4)	1024.1 (1017.4 - 1030.7)	-756.6 (-782.4 - -730.8)

CI: Confidence Interval. Adjusted by inverse probability weights on: Body mass index, smoking, weekly alcohol consumption, physical activity, disease treatment, state time arrangement, employment sector, highest educational level, and number of survey waves.

In parallel to Table E3, Table E4 shows the average annual cost per employee from an ELMA analysis with Cohen's four-item stress as the explaining variable while including all weights. Compared to the results of Table 3, the total average cost of work absenteeism is increased by 80% for men and by 202% for women. The average cost of sickness absence is doubled for men and decreased by 29% for women.

Table E4 - Annual standardized average costs in EUR of work absenteeism per employee with Cohen four-item stress by sex and age group when compared to employees without Cohen four-item stress – including the contribution of sickness absence, unemployment, and temporary out. All in 2022 price level.

Cohen four-item stress	Work absenteeism	Sickness absence	Unemployment	Temporary out
	Average EUR per employee per year (95% CI)	Average EUR per employee per year (95% CI)	Average EUR per employee per year (95% CI)	Average EUR per employee per year (95% CI)
Men				
18-34 years	15143.1 (15047.8 - 15238.4)	1872.4 (1858.9 - 1885.9)	1636.2 (1617.8 - 1654.7)	-7013.6 (-7073.9 - -6953.3)
35-49 years	774.0 (737.8 - 810.1)	2468.5 (2455.6 - 2481.5)	36.9 (30.3 - 43.4)	-1839.6 (-1853.2 - -1825.9)
50-64 years	-3710.7 (-3785.8 - -3635.7)	2566.9 (2546.5 - 2587.3)	1680.6 (1667.9 - 1693.4)	526.2 (522.8 - 529.7)
Total	3432.7 (3371.0 - 3494.4)	2331.5 (2322.2 - 2340.7)	986.4 (977.8 - 995.1)	-2567.9 (-2593.8 - -2541.9)
Women				
18-34 years	23930.4 (23815.3 - 24045.4)	-2780.8 (-2807.9 - -2753.7)	821.6 (809.5 - 833.6)	-11410.5 (-11478.1 - -11342.9)
35-49 years	7490.8 (7455.3 - 7526.3)	4652.8 (4634.1 - 4671.5)	950.7 (946.8 - 954.7)	114.7 (105.2 - 124.3)
50-64 years	7556.3 (7505.5 - 7607.2)	1944.7 (1927.8 - 1961.5)	1789.4 (1780.2 - 1798.6)	385.6 (383.6 - 387.5)
Total	11810.1 (11756.4 - 11863.9)	1844.2 (1824.3 - 1864.2)	1184.8 (1179.7 - 1190.0)	-2812.3 (-2845.2 - -2779.3)

CI: Confidence Interval. Adjusted by inverse probability weights on: Body mass index, smoking, weekly alcohol consumption, physical activity, disease treatment, state time arrangement, employment sector, highest educational level, and number of survey waves.

In addition to Table E3 and Table E4, Table E5 moreover, shows the average annual cost per employee from an ELMA analysis with Job-strain as the explaining stress indicator variable while including all weights. Compared to the results of Table 3, the total average cost of work absenteeism is reduced markedly below zero for men and increased by 263% for women. The average cost of sickness absence is almost identical for men but reduced below zero for women.

Table E5 - Annual standardized average costs in EUR of work absenteeism per employee with Job-Strain by sex and age group when compared to employees without Job-Strain – including the contribution of sickness absence, unemployment, and temporary out. All in 2022 price level.

Job-strain	Work absenteeism	Sickness absence	Unemployment	Temporary out
	Average EUR per employee per year (95% CI)	Average EUR per employee per year (95% CI)	Average EUR per employee per year (95% CI)	Average EUR per employee per year (95% CI)
Men				
18-34 years	219.1 (72.9 - 365.3)	933.6 (919.1 - 948.1)	-367.8 (-377.7 - -357.9)	-7930.2 (-7997.3 - -7863.1)
35-49 years	-4040.5 (-4083.1 - -3998.0)	3156.4 (3138.1 - 3174.7)	634.4 (628.7 - 640.2)	2889.1 (2874.9 - 2903.3)
50-64 years	-2966.5 (-3062.6 - -2870.3)	-1563.9 (-1588.3 - -1539.4)	3065.9 (3051.2 - 3080.5)	-116.4 (-120.3 - -112.4)
Total	-2695.9 (-2747.0 - -2644.7)	1143.1 (1124.6 - 1161.6)	1164.4 (1153.2 - 1175.5)	-613.8 (-648.3 - -579.3)
Women				
18-34 years	18797.6 (18638.9 - 18956.3)	997.2 (966.0 - 1028.5)	571.8 (559.1 - 584.4)	-13011.9 (-13088.4 - -12935.4)
35-49 years	11679.0 (11628.7 - 11729.4)	-3060.4 (-3080.1 - -3040.6)	912.4 (903.7 - 921.1)	-46.4 (-60.7 - -32.2)
50-64 years	1792.3 (1741.8 - 1842.9)	1582.6 (1555.6 - 1609.5)	305.0 (296.0 - 314.1)	-59.1 (-61.1 - -57.1)
Total	10292.0 (10227.9 - 10356.0)	-454.7 (-474.4 - -435.0)	621.8 (615.8 - 627.7)	-3474.4 (-3515.3 - -3433.5)

CI: Confidence Interval. Adjusted by inverse probability weights on: Body mass index, smoking, weekly alcohol consumption, physical activity, disease treatment, state time arrangement, employment sector, highest educational level, and number of survey waves.

Comparing Table 3 with the results across the three sensitivity analyses of the individual stress indicators, we see that women have an increased total average cost of work absenteeism and a decreased total average cost of sickness absence when including only the single indicators. For men the results on work absenteeism are less clear, however, the cost of sickness absence is generally higher when using only a single stress indicator.

Sensitivity analysis on part-time and crude estimates

The main analysis contained only standardized wages (37-hour working week). However, the Danish labor market contains a large group of part-time employees with an hourly working time of less than 37 hours. For this reason, we conducted a sensitivity analysis in which the part-time employees from the sample kept their part-time wages. Table E6 shows the results for employees with part-time wages included. For employees with a workweek exceeding 37 hours, we kept the standardized 37-hour wage for two reasons: (i) uncertainty concerning the permanent nature of the exceeded working time, (ii) wage payments during sickness absence are typically limited to 7.4 hours per day (37 hours per week) and do not include any extra working time.

Table E6 – ELMA results converted to annual average costs of work absenteeism per standardized full-time employee and non-standardized part-time employee - from increased work-stress levels, by sex and age group – and the contribution of sickness absence, unemployment, and temporary out. All in EUR 2022 price level.

Number of work-stress indicators	Work absenteeism	Sickness absence	Unemployment	Temporary out
	Average EUR per employee per year (95% CI)	Average EUR per employee per year (95% CI)	Average EUR per employee per year (95% CI)	Average EUR per employee per year (95% CI)
Men				
18-34 years				
1 of 3	497.0 (486.6 - 507.4)	-228.2 (-233.5 - -222.9)	545.5 (541.9 - 549.1)	470.4 (461.7 - 479.2)
2 of 3	1503.4 (1486.1 - 1520.7)	2122.7 (2113.8 - 2131.5)	1723.9 (1717.9 - 1729.9)	-3172.6 (-3187.2 - -3158.1)
3 of 3	-4173.1 (-4204.2 - -4142.1)	-217.1 (-233.0 - -201.2)	297.9 (287.1 - 308.7)	-5917.6 (-5943.7 - -5891.5)
35-49 years				
1 of 3	1128.6 (1123.5 - 1133.6)	502.3 (498.6 - 505.9)	90.3 (88.7 - 92.0)	4.5 (2.6 - 6.4)
2 of 3	2480.8 (2472.8 - 2488.9)	1064.6 (1058.8 - 1070.4)	350.5 (347.9 - 353.2)	431.1 (428.0 - 434.2)
3 of 3	6883.1 (6871.7 - 6894.4)	4859.5 (4851.3 - 4867.7)	539.7 (536.0 - 543.4)	1083.4 (1079.0 - 1087.7)
50-64 years				
1 of 3	1223.0 (1216.2 - 1229.7)	588.1 (584.8 - 591.4)	1029.5 (1024.9 - 1034.1)	167.6 (165.3 - 169.9)
2 of 3	5642.7 (5631.8 - 5653.5)	3655.8 (3650.5 - 3661.1)	2424.6 (2417.3 - 2432.0)	1199.2 (1195.5 - 1202.9)
3 of 3	5361.4 (5342.9 - 5379.9)	4229.0 (4219.9 - 4238.1)	4918.7 (4906.1 - 4931.3)	25.8 (19.5 - 32.2)
Total	1858.7 (1848.3 - 1869.2)	1115.6 (1108.3 - 1122.9)	817.8 (812.8 - 822.7)	-37.8 (-44.4 - -31.3)
Women				
18-34 years				
1 of 3	1956.9 (1944.4 - 1969.3)	1560.5 (1553.5 - 1567.4)	347.6 (343.6 - 351.7)	-167.6 (-173.9 - -161.4)
2 of 3	1915.9 (1898.8 - 1933.1)	944.3 (934.7 - 953.9)	1764.4 (1758.8 - 1770.0)	-2188.8 (-2197.4 - -2180.1)
3 of 3	8208.1 (8180.5 - 8235.7)	4689.1 (4673.7 - 4704.6)	1605.1 (1596.1 - 1614.2)	98.3 (84.5 - 112.2)
35-49 years				
1 of 3	2502.2 (2496.5 - 2507.9)	1467.1 (1464.2 - 1469.9)	213.5 (211.0 - 216.0)	214.7 (211.5 - 217.9)
2 of 3	6531.0 (6522.8 - 6539.2)	3174.2 (3170.1 - 3178.2)	1452.1 (1448.5 - 1455.7)	2696.0 (2691.4 - 2700.7)
3 of 3	10321.3 (10308.5 - 10334.1)	6405.5 (6399.1 - 6411.9)	4253.3 (4247.7 - 4258.9)	876.3 (869.1 - 883.6)
50-64 years				
1 of 3	2162.0 (2157.3 - 2166.6)	1911.3 (1908.1 - 1914.4)	389.3 (388.0 - 390.6)	171.3 (170.2 - 172.4)
2 of 3	4780.9 (4774.2 - 4787.5)	4128.6 (4124.1 - 4133.1)	952.6 (950.7 - 954.4)	187.9 (186.4 - 189.4)
3 of 3	5977.9 (5966.9 - 5988.9)	5388.8 (5381.4 - 5396.2)	1283.2 (1280.1 - 1286.3)	507.4 (504.9 - 510.0)
Total	3708.7 (3698.3 - 3719.0)	2480.6 (2474.2 - 2487.0)	749.4 (745.3 - 753.5)	267.4 (262.7 - 272.1)

The Total estimate uses a standardized weighted average. CI: Confidence Interval. Adjusted by inverse probability weights on: Body mass index, Smoking, weekly alcohol consumption, physical activity, disease treatment, state time arrangement, employment sector, highest educational level, and number of survey waves.

Table E6 shows that the inclusion of part-time wages reduces the average cost estimates by a small fraction when compared to the cost concerning all 37-hourly standardized wages (Table 3). In particular, the cost of

work absenteeism is lower in Table E6 than in Table 3 - for men and women, and across age groups and the number of work-stress indicators.

Additionally, we conducted the cost analysis by using the crude estimate of work absenteeism, sickness absence, unemployment, and temporary out (Table E7). This was done to compare with the respective costs based on the ELMA estimates.

Table E7 - Crude results converted to annual standardized (37-hours per week) cumulated costs of work absenteeism per full-time employee from increased work-stress levels, by sex and age group – and the contribution of sickness absence, unemployment, and temporary out. All in EUR 2022 price level.

	Work absenteeism	Sickness absence	Unemployment	Temporary out
Number of work-stress indicators	EUR per employee per year	EUR per employee per year	EUR per employee per year	EUR per employee per year
Men				
18-34 years				
1 of 3	111.2	395.3	345.9	-617.7
2 of 3	1874.9	1589.3	1465.2	-1167.2
3 of 3	317.6	965.1	1637.0	-2272.3
35-49 years				
1 of 3	481.8	642.4	16.1	-128.5
2 of 3	3717.2	1731.5	524.2	1509.1
3 of 3	5779.9	3194.2	745.3	1308.1
50-64 years				
1 of 3	-46.4	897.1	587.7	154.7
2 of 3	1123.1	2198.7	1645.0	316.4
3 of 3	-1914.8	3053.8	1535.1	-82.5
Total	973.7	1153.6	563.2	-13.8
Women				
18-34 years				
1 of 3	1735.8	953.6	235.7	557.2
2 of 3	2459.0	2097.0	1139.0	-766.4
3 of 3	7912.0	3812.7	1630.9	2490.4
35-49 years				
1 of 3	2262.5	1693.6	304.3	344.0
2 of 3	5795.6	3987.8	1183.0	731.1
3 of 3	7224.3	5398.4	1230.5	714.5
50-64 years				
1 of 3	1387.8	1481.3	360.3	80.1
2 of 3	3613.1	3705.7	622.0	105.9
3 of 3	4581.2	5788.9	1180.0	360.9
Total	3092.8	2446.0	554.0	327.9

The Total uses a standardized weighted estimate.

Moreover, we made estimates using the crude method with part-time wages included (Table E8).

Table E8 - Crude results converted to annual cumulated costs of work absenteeism per standardized full-time employee and non-standardized part-time employee - from increased work-stress levels, by sex and age group – and the contribution of sickness absence, unemployment, and temporary out. All in EUR 2022 price level.

	Work absenteeism	Sickness absence	Unemployment	Temporary out
Number of work-stress indicators	EUR per employee per year	EUR per employee per year	EUR per employee per year	EUR per employee per year
Men				
18-34 years				
1 of 3	104.8	372.7	326.1	-582.4
2 of 3	1777.7	1506.9	1389.2	-1106.6
3 of 3	308.7	937.9	1590.8	-2208.2
35-49 years				
1 of 3	472.4	629.9	15.7	-126.0
2 of 3	3670.5	1709.7	517.6	1490.1
3 of 3	5645.0	3119.6	727.9	1277.5
50-64 years				
1 of 3	-45.4	877.7	575.0	151.3
2 of 3	1096.4	2146.4	1606.0	308.8
3 of 3	-1888.1	3011.2	1513.8	-81.4
Total	950.9	1125.0	546.1	-5.8
Women				
18-34 years				
1 of 3	1612.5	885.9	219.0	517.6
2 of 3	2270.5	1936.3	1051.7	-707.7
3 of 3	7325.5	3530.1	1510.0	2305.8
35-49 years				
1 of 3	2156.1	1613.9	290.0	327.8
2 of 3	5525.1	3801.6	1127.8	697.0
3 of 3	6902.5	5157.9	1175.7	682.7
50-64 years				
1 of 3	1322.8	1411.9	343.4	76.3
2 of 3	3444.0	3532.3	592.9	100.9
3 of 3	4395.1	5553.8	1132.1	346.3
Total	2931.2	2322.0	524.3	310.4

The Total uses a standardized weighted estimate.

We compared the ELMA costs estimates with the crude estimates: Table 3 versus Table E7 and Table E6 versus Table E8. The average ELMA costs on work absenteeism and sickness absence are generally

significantly higher than the respective costs of the crude method – except for sickness absence for men, which are more mixed. For unemployment and temporary out, the two types of estimates are more similar but occasionally a little lower concerning the crude method.

F - The hypothetical reduction potential regarding work absenteeism and sickness absence

We estimated the hypothetical reduction potential regarding the total annual value of work absenteeism and sickness absence. We conducted the estimations by reducing the overall work-stress level by 10%, through these three steps:

1. 10% of the employees with 3 of 3 indicators, reduced the work-stress level to 2 of 3 indicators
2. 10% of the employees with 2 of 3 indicators reduced the work-stress level to 1 of 3 indicators
3. 10% of the employees with 1 of 3 indicators reduced to no work-stress indicators.

We then conducted similar estimations at 50% and at 100% reductions.

Table F1 – Annual standardized and weighted total costs of work absenteeism associated with the increased number of work-stress indicators, by sex and age group – and the contribution of sickness absence, unemployment, and temporary out. All in EUR and 2022 price level.

Number of work-stress indicators	Weighted N (%)	Work absenteeism<br>Total million EUR per year (95% CI)	Sickness absence<br>Total million EUR per year (95% CI)	Unemployment<br>Total million EUR per year (95% CI)	Temporary out<br>Total million EUR per year (95% CI)
Men					
18-34 years					
0 of 3	102442 (71)				
1 of 3	28668 (20)	15.1 (14.8 - 15.4)	-6.9 (-7.1 - -6.8)	16.6 (16.5 - 16.7)	14.3 (14.0 - 14.6)
2 of 3	10585 (7)	16.8 (16.6 - 17.0)	23.7 (23.6 - 23.8)	19.2 (19.2 - 19.3)	-35.4 (-35.6 - -35.3)
3 of 3	3337 (2)	-14.3 (-14.4 - -14.2)	-0.7 (-0.8 - -0.7)	1.0 (1.0 - 1.1)	-20.3 (-20.4 - -20.2)
35-49 years					
0 of 3	157254 (70)				
1 of 3	43619 (19)	50.2 (50.0 - 50.4)	22.3 (22.2 - 22.5)	4.0 (3.9 - 4.1)	0.2 (0.1 - 0.3)
2 of 3	16924 (8)	42.5 (42.4 - 42.7)	18.2 (18.1 - 18.3)	6.0 (6.0 - 6.1)	7.4 (7.3 - 7.4)
3 of 3	7705 (3)	54.3 (54.2 - 54.4)	38.3 (38.3 - 38.4)	4.3 (4.2 - 4.3)	8.5 (8.5 - 8.6)
50-64 years					
0 of 3	143528 (74)				
1 of 3	31815 (16)	39.8 (39.5 - 40.0)	19.1 (19.0 - 19.2)	33.5 (33.3 - 33.6)	5.4 (5.4 - 5.5)
2 of 3	12861 (7)	74.3 (74.2 - 74.5)	48.2 (48.1 - 48.2)	31.9 (31.8 - 32.0)	15.8 (15.7 - 15.8)
3 of 3	4880 (3)	26.5 (26.4 - 26.6)	20.9 (20.9 - 21.0)	24.3 (24.3 - 24.4)	0.1 (0.1 - 0.2)
Total		305.2 (304.2 - 306.3)	183.2 (182.5 - 183.8)	140.9 (140.4 - 141.4)	-3.9 (-4.1 - -3.7)
Women					
18-34 years					
0 of 3	98639 (63)				
1 of 3	32942 (21)	69.4 (69.0 - 69.8)	55.3 (55.1 - 55.6)	12.3 (12.2 - 12.5)	-5.9 (-6.2 - -5.7)

2 of 3	16865 (11)	35.0 (34.7 - 35.3)	17.2 (17.1 - 17.4)	32.2 (32.1 - 32.3)	-40.0 (-40.1 - -39.8)
3 of 3	7037 (5)	62.4 (62.2 - 62.6)	35.6 (35.5 - 35.8)	12.2 (12.1 - 12.3)	0.7 (0.6 - 0.9)
35-49 years					
0 of 3	182990 (66)				
1 of 3	54762 (20)	143.8 (143.5 - 144.1)	84.3 (84.1 - 84.5)	12.3 (12.1 - 12.4)	12.3 (12.2 - 12.5)
2 of 3	26695 (10)	182.9 (182.7 - 183.1)	88.9 (88.8 - 89.0)	40.7 (40.6 - 40.8)	75.5 (75.4 - 75.6)
3 of 3	10910 (4)	117.9 (117.7 - 118.0)	73.1 (73.1 - 73.2)	48.6 (48.5 - 48.6)	10.0 (9.9 - 10.1)
50-64 years					
0 of 3	163337 (69)				
1 of 3	43391 (18)	98.4 (98.2 - 98.6)	87.0 (86.9 - 87.2)	17.7 (17.7 - 17.8)	7.8 (7.7 - 7.8)
2 of 3	20979 (9)	105.2 (105.1 - 105.4)	90.9 (90.8 - 91.0)	21.0 (20.9 - 21.0)	4.1 (4.1 - 4.2)
3 of 3	8587 (4)	53.5 (53.4 - 53.6)	48.2 (48.2 - 48.3)	11.5 (11.5 - 11.5)	4.5 (4.5 - 4.6)
Total		868.5 (865.9 - 871.0)	580.7 (579.0 - 582.3)	208.4 (207.8 - 209.0)	69.1 (68.8 - 69.5)

CI: Confidence Interval. Adjusted by inverse probability weights on: Body mass index, smoking, weekly alcohol consumption, physical activity, disease treatment, state time arrangement, employment sector, highest educational level, and number of survey waves.

From the results in Table 3, we estimate the annual reduction potential regarding the costs of work absenteeism and the contribution of sickness absence. For a 10% work-stress reduction, the annual reduction in costs of work absenteeism was 7% for both sexes (Men: 21.1 million EUR, Women: 58.2 million EUR), for a 50% work-stress reduction the costs of work absenteeism was reduced by 34% for women (291.4 million EUR) and 35% for men (105.6 million EUR). For the 100% work-stress reduction, the annual costs of work absenteeism were reduced by 69% for men (211.2 million EUR) and 67% for women (582.8 million EUR).

For the 10% work-stress reduction, the annual contribution of sickness absence to costs of work absenteeism was reduced by 7% for both sexes (Men: 13.5 million EUR, Women: 38.8 million EUR). For 50% work-stress reduction, the annual contribution of sickness absence was reduced by 33% for women (194.2 million EUR) and 37% for men (67.6 million EUR), and at 100% work-stress reduction, the annual contribution of sickness absence was reduced by 67% for women (388.5 million EUR) and 74% for men (135.3 million EUR).

G - Top-down estimation of society costs

Firstly, we assume that the remaining 46% of the Danish labor force is representable by the results corresponding to the study sample. Then we can estimate the annual total costs of work absenteeism linked to work-related stress of one to all three indicators, as 2.2 billion EUR ((305.3 million + 868.5 million)/ 0.54). Calculated in a similar way, the annual contribution of costs of cumulated sickness absence days associated with work stress is 1.4 billion EUR ((183.2 million + 580.7 million) / 0.54). According to

Perriard et al. (4), wages for sickness absence make up 59.9% of the total costs related to work stress in Switzerland – while the remaining elements are medical and self-medical expenses (4). Assuming somewhat arbitrarily that wages for sickness absence also constitute 59.9% of the total costs related to work stress in Denmark, the Danish total cost of work-related stress is 2.3 billion EUR per year, corresponding to an additional annual cost of 3333.8 EUR per affected employee.

By including the unadjusted wages of part-time employees, the estimated cost of work absenteeism is 2.1 billion EUR ((298.1 million + 824.0 million)/0.54), and the estimated total cost of work-related stress is 2.3 billion EUR - including medical and self-medical expenses ((179.0 million + 551.1 million) / 54.0%*59.9%).

Overall, the results of the fully adjusted 37-hour wages are approx. 0.1 billion EUR higher than the results including part-time wages.

For the analysis using the crude estimates, the estimated cost of work absenteeism is 1.6 billion EUR ((156.2 million + 687.1 million)/0.54), and the total cost of work-related stress is 2.3 billion EUR including medical and self-medical expenses ((185.0 million + 543.4 million) / 54.0%*59.9%). For the analysis including part-time wages, the estimated cost of work absenteeism is 1.5 billion EUR ((152.3 million + 651.2 million)/0.54), and the total cost of work-related stress is 2.2 billion EUR including medical and self-medical expenses ((180.4 million + 515.9 million) / 54.0%*59.9%).

Again, the results of the fully adjusted 37-hour wages are approx. 0.1 billion EUR higher than the results including part-time wages. However, the crude results on the cost of work absenteeism are approx. 0.6 billion EUR lower than the ELMA results, while the results based on sickness absence costs are approx. 0.1 billion lower than the ELMA estimates.

The Crude-estimates are the summarized number of days by state divided by the total number of employees. The Crude-estimate does not include any consideration of the risk-time of the individual employee – for example, it can affect the estimate if a number of employees do not have a complete two-year follow-up time. Moreover, for the crude method, the order of events is not important as all events may as well happen sporadically over time as they may happen simultaneously in time – like periods of sickness absence within time of work. However, the order of events is very important when using a survival approach – as many simultaneous events of e.g. sick-listing will drastically increase the risk of sickness absence at a particular time.

Since most of the study follow-up time contains work periods, the crude method is particularly likely to underestimate the expected duration of work when follow-up periods are truncated frequently while the

employees are working. That is why the difference between the ELMA results and the Crude-estimates is particularly large for the duration of work time and less different, for periods of sickness absence etc.

H – Disability pension, retirement, and death

Table H1 - The ELMA results given by the expected average number of days during the two-year follow-up period spent in the four recurrent labor market states stratified by gender and age groups. Reference value showing the expected days and the additional or subtracted days (+/-) for employees with 1, 2, or 3 indicators of work stress. The ELMA model estimates and the crude unadjusted estimates.

Number of work-stress indicators	Disability pension		Retirement		Death	
	ELMA	Crude	ELMA	Crude	ELMA	Crude
	Days (95% CI) per 2-year	Days per 2-year	Days (95% CI) per 2-year	Days per 2-year	Days (95% CI) per 2-year	Days per 2-year
Men						
18-34 years						
Ref. 0 of 3						
1 of 3						
2 of 3						
3 of 3						
35-49 years						
Ref. 0 of 3		0.2			0.2 (0.2 - 0.3)	0.2
1 of 3					-0.2 (-0.3 - -0.2)*	
2 of 3					-0.2 (-0.3 - -0.2)*	
3 of 3					-0.2 (-0.3 - -0.2)*	+3.5
50-64 years						
Ref. 0 of 3	0.2 (0.1 - 0.3)	0.5	32.5 (30.2 - 34.9)	63.0	0.4 (-0.1 - 0.8)	1.5
1 of 3	-0.1 (-0.3 - 0.0)	-0.2	-7.5 (-10.8 - -4.3)*	-11.1	0.0 (-0.6 - 0.6)	+0.5
2 of 3	+0.3 (0.1 - 0.5)*	-0.2	-15.6 (-18.9 - -12.3)*	-19.6	+1.4 (0.8 - 2.0)*	+0.7
3 of 3	-0.1 (-0.3 - 0.0)	+1.2	-26.4 (-29.7 - -23.2)*	-38.6	0.0 (-0.6 - 0.7)	-1.5
Women						
18-34 years						
Ref. 0 of 3		0.1				
1 of 3						
2 of 3						
3 of 3						
35-49 years						
Ref. 0 of 3	0.1 (0.0 - 0.2)	0.4			0.2 (0.1 - 0.2)	0.4
1 of 3	0.0 (-0.1 - 0.2)				-0.2 (-0.2 - -0.1)*	
2 of 3	+0.1 (-0.1 - 0.2)	-0.3			-0.2 (-0.2 - -0.1)*	
3 of 3	-0.1 (-0.2 - 0.0)				-0.2 (-0.2 - -0.1)*	
50-64 years						
Ref. 0 of 3	0.3 (0.0 - 0.7)	1.0	26.6 (24.4 - 28.9)	67.3	0.3 (0.0 - 0.6)	1.3
1 of 3	+1.4 (0.9 - 1.8)*	+0.7	-4.2 (-7.4 - -1.0)*	-4.0	-0.2 (-0.6 - 0.3)	-0.7
2 of 3	+0.5 (0.1 - 1.0)	-0.5	-5.9 (-9.1 - -2.7)*	-6.4	+0.5 (0.0 - 0.9)	+0.7
3 of 3	+0.4 (-0.1 - 0.9)	-0.6	-10.2 (-13.4 - -7.0)*	-18.7	+0.4 (-0.1 - 0.8)	-0.4

* 5% significant, Ref.: Reference value, CI: Confidence Interval. ELMA results are adjusted by inverse probability weights on: Body mass index, smoking, weekly alcohol consumption, physical activity, disease treatment, state time arrangement, employment sector, highest educational level, and number of survey waves.

I – Output from the multi-state Cox-regression model

The ELMA method is based on the multi-state survival method, which implies that all hazard ratios are based on transition-specific Cox regressions each with a specific baseline hazard. The Cox regression may be conducted on each transition one at a time or simultaneously if the data is arranged in a "long format". Table I1 contains the hazard ratios for the transitions between the states of work, sickness absence, and unemployment. The hazard ratios of the remaining transitions are not shown, e.g. between work and retirement, sickness absence and disability pension, temporary out and work, etc.

Table I1 - Hazard ratios and 95% confidence intervals for the transitions between the work, sickness absence, and unemployment state - during the two-year follow-up period and adjusted by inverse probability weights (see footnote). Separate analyses were made on gender and age groups.

Number of work-stress indicators	Men			Women		
	18-34 years	35-49 years	50-64 years	18-34 years	35-49 years	50-64 years
	HR (95% CI)	HR (95% CI)	HR (95% CI)	HR (95% CI)	HR (95% CI)	HR (95% CI)
Work to Sickness absence						
0 of 3	1.00 (-)	1.00 (-)	1.00 (-)	1.00 (-)	1.00 (-)	1.00 (-)
1 of 3	1.09 (0.91-1.31)	1.14 (1.03-1.27)**	1.12 (1.02-1.24)*	1.27 (1.13-1.42)**	1.17 (1.09-1.26)**	1.26 (1.17-1.36)**
2 of 3	0.93 (0.75-1.15)	1.24 (1.09-1.43)**	1.31 (1.14-1.51)**	1.20 (1.03-1.41)*	1.32 (1.20-1.45)**	1.48 (1.37-1.60)**
3 of 3	1.11 (0.85-1.46)	1.28 (1.01-1.63)*	1.40 (1.18-1.67)**	1.20 (0.99-1.46)	1.44 (1.30-1.60)**	1.44 (1.30-1.59)**
Work to Unemployment						
0 of 3	1.00 (-)	1.00 (-)	1.00 (-)	1.00 (-)	1.00 (-)	1.00 (-)
1 of 3	6.68 (3.00-14.85)**	0.68 (0.31-1.51)	2.14 (0.81-5.63)	1.60 (0.77-3.32)	3.60 (1.29-10.04)*	1.46 (0.81-2.63)
2 of 3	4.22 (0.99-17.98)	0.92 (0.23-3.69)	1.06 (0.53-2.13)	2.74 (0.91-8.25)	1.03 (0.61-1.74)	0.91 (0.43-1.91)
3 of 3	0.09 (0.01-0.70)*	0.68 (0.25-1.82)	1.89 (0.40-8.99)	2.36 (0.83-6.72)	2.29 (0.83-6.35)	3.43 (0.84-14.04)
Sickness absence to Work						
0 of 3	1.00 (-)	1.00 (-)	1.00 (-)	1.00 (-)	1.00 (-)	1.00 (-)
1 of 3	1.03 (0.81-1.32)	0.83 (0.68-1.00)*	0.86 (0.71-1.05)	0.91 (0.74-1.11)	0.77 (0.59-0.99)*	0.80 (0.66-0.96)*
2 of 3	0.46 (0.27-0.80)**	0.77 (0.62-0.95)*	0.56 (0.40-0.78)**	0.68 (0.54-0.85)**	0.65 (0.54-0.78)**	0.67 (0.57-0.79)**
3 of 3	1.03 (0.44-2.43)	0.36 (0.19-0.68)**	0.59 (0.38-0.90)*	0.38 (0.17-0.84)*	0.45 (0.28-0.71)**	0.55 (0.45-0.67)**
Sickness absence to Unemployment						
0 of 3	1.00 (-)	1.00 (-)	1.00 (-)	1.00 (-)	1.00 (-)	1.00 (-)
1 of 3	1.46 (0.37-5.68)	1.03 (0.43-2.50)	4.20 (1.65-10.70)**	1.41 (0.70-2.82)	1.53 (0.88-2.65)	1.07 (0.62-1.87)
2 of 3	2.42 (0.73-7.98)	1.08 (0.29-4.03)	3.67 (1.48-9.09)**	1.90 (0.78-4.60)	1.73 (1.02-2.92)*	1.26 (0.70-2.27)
3 of 3	14.13 (3.14-63.68)**	2.15 (0.66-6.96)	2.60 (0.73-9.22)	1.59 (0.51-4.96)	0.93 (0.35-2.49)	1.28 (0.57-2.88)
Unemployment to Work						
0 of 3	1.00 (-)	1.00 (-)	1.00 (-)	1.00 (-)	1.00 (-)	1.00 (-)
1 of 3	3.10 (1.47-6.56)**	0.60 (0.26-1.39)	0.84 (0.41-1.74)	1.04 (0.39-2.78)	1.99 (0.90-4.43)	1.05 (0.54-2.06)
2 of 3	1.11 (0.23-5.41)	0.60 (0.12-2.99)	0.09 (0.03-0.25)**	1.31 (0.42-4.11)	0.31 (0.14-0.66)**	0.34 (0.11-1.09)
3 of 3	0.12 (0.01-1.49)	0.11 (0.02-0.59)*	0.02 (0.00-0.21)**	0.49 (0.15-1.56)	0.06 (0.01-0.25)**	1.23 (0.25-6.16)
Unemployment to Sickness absence						
0 of 3	1.00 (-)	1.00 (-)	1.00 (-)	1.00 (-)	1.00 (-)	1.00 (-)
1 of 3	0.16 (0.03-0.94)*	1.49 (0.54-4.12)	4.24 (1.77-10.17)**	1.33 (0.69-2.57)	0.89 (0.50-1.58)	0.97 (0.58-1.62)
2 of 3	1.80 (0.39-8.37)	1.00 (0.15-6.60)	2.30 (0.93-5.72)	1.32 (0.54-3.19)	1.11 (0.62-2.02)	1.38 (0.77-2.46)
3 of 3	8.03 (2.12-30.41)**	4.69 (1.36-16.13)*	1.84 (0.31-10.85)	2.80 (1.20-6.55)*	0.40 (0.14-1.12)	1.28 (0.60-2.75)

*: 5% significant. **: 1% significant. HR: Hazard Ratio. CI: Confidence interval. Adjusted by inverse probability weights on: Body mass index, smoking, weekly alcohol consumption, physical activity, disease treatment, state time arrangement, employment sector, highest educational level, and number of survey waves.

References

1. Johnsen NF, Thomsen BL, Hansen JV, Christensen BS, Rugulies R, Schlünssen V. Job type and other socio-demographic factors associated with participation in a national, cross-sectional study of Danish employees. BMJ Open. 2019;9(8):e027056. doi: 10.1136/bmjopen-2018-027056
2. Karasek R, Choi B, Ostergren P-O, Ferrario M, Smet PD. Testing two methods to create comparable scale scores between the job content questionnaire (JCQ) and JCQ-like questionnaires in the European JACE study. International Journal of Behavioral Medicine. 2007;14(4):189-201. doi: 10.1007/BF03002993
3. Union based unemployment insurance [Accessed 2023 07-03]. Available from: https://www.a-kasser.dk/stigende-andel-af-de-unge-er-medlem-af-en-a-kasse/index.html
4. State Secretariat for Economic Affairs (SECO). Ramaciotti D, Perriard J. Die Kosten von Stress in der Schweiz [Accessed 2022 17-08]. Available from: https://www.seco.admin.ch/seco/de/home/Publikationen_Dienstleistungen/Publikationen_und_Formulare/Arbeit/Arbeitsbedingungen/Studien_und_Berichte/die-kosten-des-stresses-in-der-schweiz.html
